# Early involvement of cellular stress and inflammatory signals in the pathogenesis of tubulointerstitial kidney disease due to *UMOD* mutations

**DOI:** 10.1038/s41598-017-07804-6

**Published:** 2017-08-07

**Authors:** Matteo Trudu, Celine Schaeffer, Michela Riba, Masami Ikehata, Paola Brambilla, Piergiorgio Messa, Filippo Martinelli-Boneschi, Maria Pia Rastaldi, Luca Rampoldi

**Affiliations:** 1 Molecular Genetics of Renal Disorders Unit, Division of Genetics and Cell Biology, IRCCS San Raffaele Scientific Institute, Milan, Italy; 2 Centre for Translational Genomics and Bioinformatics, IRCCS San Raffaele Scientific Institute, Milan, Italy; 3 Fondazione IRCCS Cà Granda, Ospedale Maggiore Policlinico, Milan, Italy; 4 Università degli Studi di Milano, Milan, Italy; 5 Inflammatory CNS disorders, INSPE Unit, Division of Neuroscience, IRCCS San Raffaele Scientific Institute, Milan, Italy

## Abstract

Autosomal dominant tubulointerstitial kidney disease (ADTKD) is an inherited disorder that causes progressive kidney damage and renal failure. Mutations in the *UMOD* gene, encoding uromodulin, lead to ADTKD-*UMOD* related. Uromodulin is a GPI-anchored protein exclusively produced by epithelial cells of the thick ascending limb of Henle’s loop. It is released in the tubular lumen after proteolytic cleavage and represents the most abundant protein in human urine in physiological condition. We previously generated and characterized a transgenic mouse model expressing mutant uromodulin (Tg^*Umod*C147W^) that recapitulates the main features of ATDKD-*UMOD*. While several studies clearly demonstrated that mutated uromodulin accumulates in endoplasmic reticulum, the mechanisms that lead to renal damage are not fully understood. In our work, we used kidney transcriptional profiling to identify early events of pathogenesis in the kidneys of Tg^*Umod*C147W^ mice. Our results demonstrate up-regulation of inflammation and fibrosis and down-regulation of lipid metabolism in young Tg^*Umod*C147W^ mice, before any functional or histological evidence of kidney damage. We also show that pro-inflammatory signals precede fibrosis onset and are already present in the first week after birth. Early induction of inflammation is likely relevant for ADTKD-*UMOD* pathogenesis and related pathways can be envisaged as possible novel targets for therapeutic intervention.

## Introduction

Tubulointerstitial kidney diseases occur with a diverse array of causes, including genetic disorders, and constitute an important cause of chronic kidney disease (CKD). Inherited renal tubulointerstitial disorders include autosomal dominant tubulointerstitial kidney disease (ADTKD), characterised by interstitial fibrosis with tubular atrophy and dilation, and thickening and lamellation of tubular basal membranes^1^. Five ADTKD genes have been identified so far: *UMOD* (16p12)^2^, *MUC1* (mucin 1, 1q21)^3^, *HNF1B* (HNF1beta, 17q12)^4^, *REN* (renin, 1q32)^5^ and *SEC 61A1* (Sec 61 translocon alpha 1 subunit, 3q21)^6^. ADTKD-*UMOD* patients reach end-stage renal disease between 20 and 70 years of age. Currently, no specific treatment can be offered other than renal replacement therapy. The *UMOD* gene encodes uromodulin, the most abundant protein in human urine in physiological condition. Uromodulin is a highly glycosylated protein that is exclusively produced by epithelial cells lining the thick ascending limb of Henle’s loop (TAL) and released in the tubular lumen after cleavage mediated by the serine protease hepsin^7^. To date, over 100 *UMOD* mutations have been described. ADTKD-*UMOD* (MIM 162000, 603860, 191845) is typically characterised by decreased fractional excretion of urate, causing hyperuricemia and often gout^8^. The biological function of uromodulin is still not fully understood. Studies in *Umod* knock-out mice showed that it has a protective role against urinary tract infections and calcium oxalate crystals damage^9, 10^. Uromodulin was shown to regulate ion transport in the TAL^11, 12^. It has been proposed to act as a kidney-specific damage associated molecular pattern that can activate interstitial dendritic cells when released in the interstitium^13, 14^. Also, uromodulin was shown to protect renal tubules from ischemia reperfusion injury^15^.

Several genome-wide association studies identified common variants in the *UMOD* gene promoter associated with increased risk of developing hypertension and CKD^16, 17^ by having an effect on *UMOD* expression and consequent urinary protein levels^12, 18^.

We and others demonstrated that *UMOD* mutations lead to defective trafficking to the plasma membrane and endoplasmic reticulum (ER) retention of mutant uromodulin^19, 20^. This is consistent with findings in patient renal biopsies, typically showing the presence of large intracellular aggregates of uromodulin in TAL epithelial cells and abnormal expansion of ER stacks^21^ and with reduced uromodulin levels in patient urines^22–24^. In our laboratory, we generated a transgenic mouse expressing C147W mutant uromodulin (Tg^*Umod*C147W^) (corresponding to patient mutation C148W)^25^. Tg^*Umod*C147W^ mice specifically show progressive signs of renal damage, i.e. tubulointerstitial fibrosis with inflammatory cell infiltration and tubule dilation, urinary concentrating defect and renal failure. These features are associated with early ER retention and aggregation of uromodulin. A similar phenotype was described in three additional mouse models, carrying different *Umod* missense mutations (A227T, C93F, C125R)^26–28^. Altogether, studies in mouse models clearly establish a main gain-of-function effect of uromodulin mutations, also supported by the fact that *Umod* knock-out mice do not develop histological features of ADTKD-*UMOD* ^29^. Since signs of renal failure and urine concentrating defect have been reported in mice lacking uromodulin^30^, we cannot exclude a partial loss-of-function component, possibly due to sequestration of wild type uromodulin in aggregates.

The cellular pathways leading to the clinical manifestations of the disease are currently unknown. To gain insight into ADTKD-*UMOD* pathophysiology, and in particular to study the early events that precede signs of renal disease, we carried out transcriptional profiling of kidneys from young, pre-symptomatic Tg^*Umod*C147W^ mice. Our results show early induction of inflammatory signals that precede fibrotic pathways and renal damage, and that likely play a key role in the disease onset.

## Results

### Transcriptional profiling of kidneys from Tg^*Umod*C147W^ and Tg^*Umod*wt^ mice

We previously showed that Tg^*Umod*C147W^ mice already display mild tubulointerstitial damage at 3 months of age, and that renal damage progresses into severe interstitial fibrosis, inflammatory cell infiltrate, tubular atrophy and renal failure by 6 months. Up to this time point Tg^*Umod*wt^ mice, i.e. transgenic mice for wild type uromodulin serving as an expression-matched control, were similar to control, non-transgenic mice^25^.

**Figure 1:**
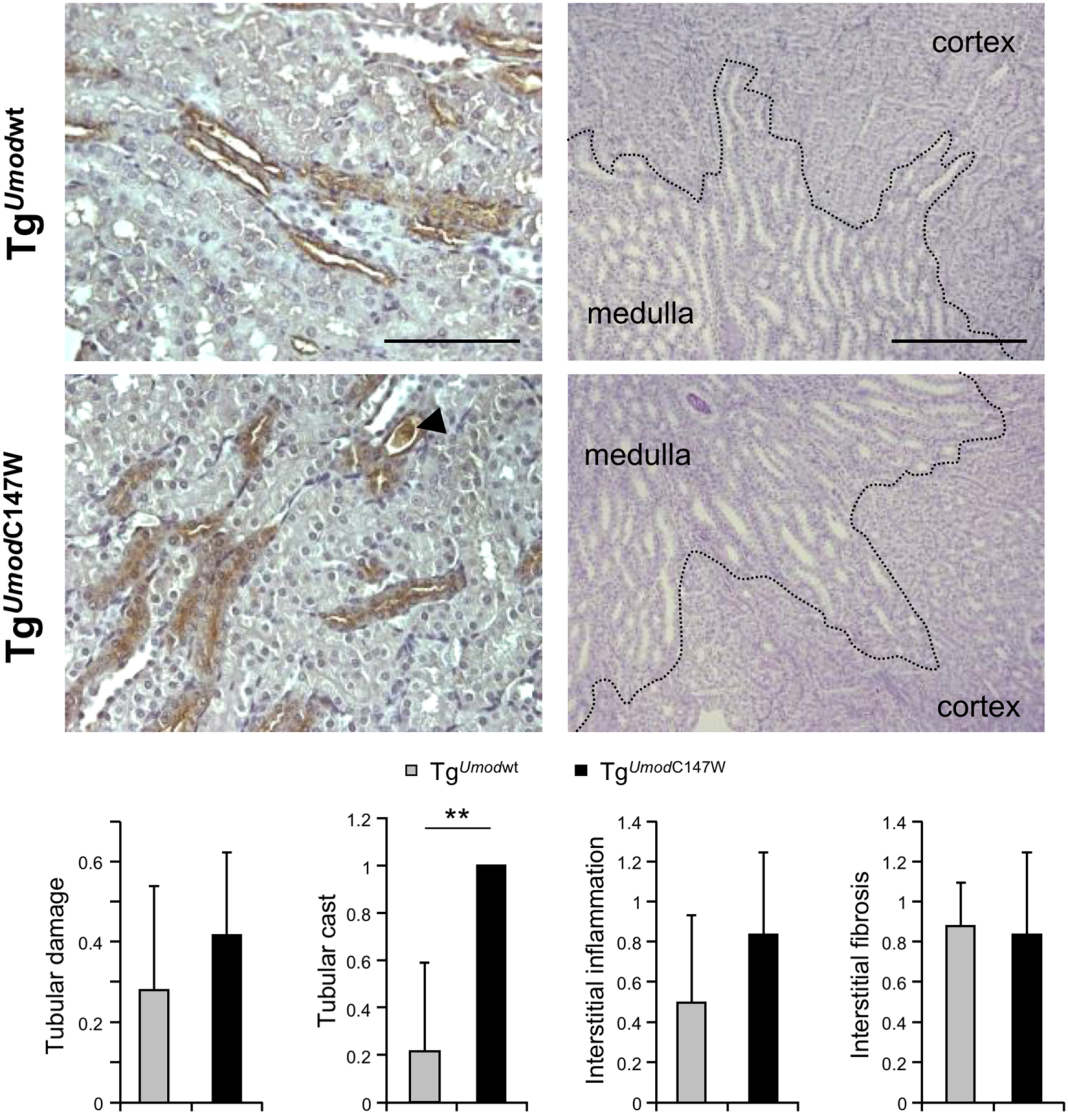
Representative images of kidneys from 1 month-old Tg^*Umod*C147W^ and Tg^*Umod*wt^ mice. Immunohistochemistry analysis for total uromodulin shows that the protein is mainly distributed at the apical membrane of TAL cells in Tg^*Umod*wt^ mice, while it is intracellularly enriched in Tg^*Umod*C147W^ mice (left panels, scale bar 100 µm). Representative images of kidney sections (right panels, PAS, scale bar 200 µm) and quantification of histological parameters (bottom) are shown. Data are expressed as mean ± s.d. (n = 9 Tg^*Umod*wt^ and 6 Tg^*Umod*C147W^). Tg^*Umod*C147W^ mice show a relatively well preserved kidney structure. Only tubular casts, mostly uromodulin-positive (left panel, arrowhead) resulted to be increased. **P < 0.01 (Mann-Whitney test).

**Figure 2:**
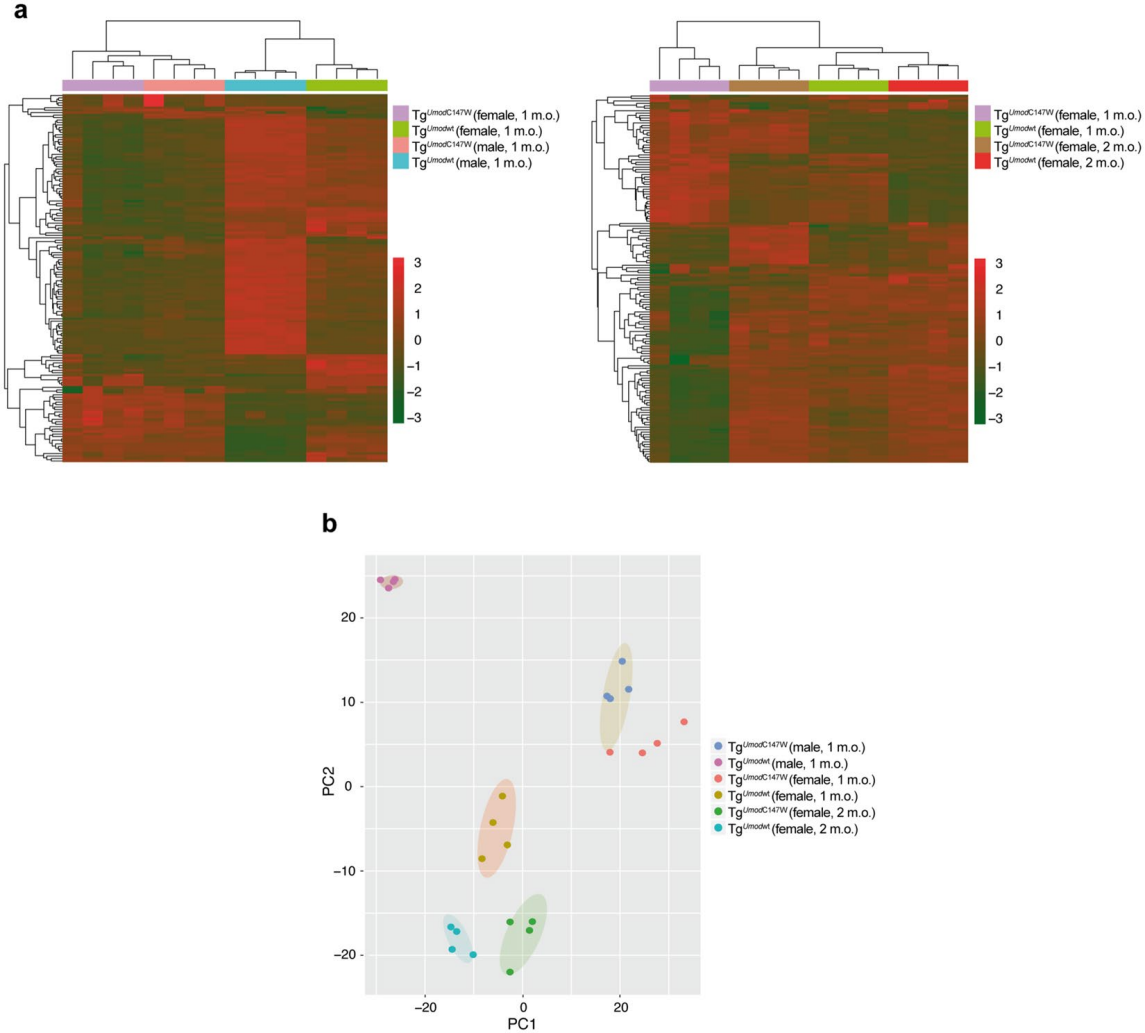
Multivariate Analysis and Principal Component Analysis (PCA) of experimental samples and groups. (**a**) The figure shows hierarchical clustering on the 150 genes showing the greatest inter-group variance among expressed genes in mice at 1 month of age (left panel) or in female mice only (right panel). (**b**) PCA based on the expression values of the 17,200 genes detected in the experimental samples (detection *P* value < 0.01 in at least one sample of an analysed series). A bi-dimensional visualization of the first two principal components is shown (PC1 accounted for 35.2% and PC2 for 25% of the variance among samples).

Aiming at identifying early events triggered by the expression of mutant uromodulin, we performed transcriptional profiling of kidneys from 1 month-old Tg^*Umod*C147W^ and Tg^*Umod*wt^ mice (males and females). At this age mutant uromodulin is already strongly accumulating in the ER of TAL cells in the kidneys of Tg^*Umod*C147W^ mice (Supplementary Figure 1), while no renal damage can be observed at the histological level, except for an increase in the number of tubular casts formation (Fig. 1 and Supplementary Figure 2). We also carried out transcriptional profile analysis on kidneys from female mice at a later time point (2 month-old), with the goal of identifying pathways related to the progression of the disease. In kidneys from 1 month-old mice we obtained positive hybridisation signal for 19,026 (males) and 18,523 (females) probes, corresponding to 13,662 and 13,357 genes expressed in males and females respectively. At 2 months of age 18,302 probes, corresponding to 13,187 genes, were expressed. Multivariate analyses performed on expressed genes using hierarchical clustering and Principal Component Analysis (PCA) allowed the separation of Tg^*Umod*C147W^ sample groups from Tg^*Umod*wt^ ones (Fig. 2). This demonstrates that mutant uromodulin expression is inducing significant transcriptional changes in the kidney at a time point in which renal structure is still preserved. Differential expression analysis was assessed in each of the 3 age- and sex-matched comparisons between Tg^*Umod*C147W^ and Tg^*Umod*wt^ mice, using a cut-off adjusted *P* value 0.05 and fold change 2. The number of genes up-regulated in 1 month-old Tg^*Umod*C147W^ mice were 92 and 68 while the down-regulated ones were 179 and 89 in males and females respectively. In the 2 month-old Tg^*Umod*C147W^ mice we found 41 up-regulated and 15 down-regulated genes (Table 1). We validated results from the microarray analysis by real-time RT-qPCR, selecting genes that were differentially expressed (adjusted *P* < 0.05) in all comparisons. In particular, we chose transcripts highly (fold change >  ± 2; *Col1a1* and *Slc25a17*), moderately (fold change ~ ±1.5; *Ntn4* and *Slc13a3*) or only slightly (fold change ~ +1.1; *Panx1*) up- or down-regulated in Tg^*Umod*C147W^ mice. Differential expression was confirmed for all analysed genes (Table 2).

**Table 1:** Genes differentially expressed in kidneys of Tg^*Umod*C147W^ mice relative to age- and sex-matched Tg^*Umod*wt^ mice.

	Genes up-regulated in Tg^*Umod*C147W^	Genes down-regulated in Tg^*Umod*C147W^
**Males (1 m**.**o**.**)**	92	179
**Females (1 m**.**o**.**)**	68	89
**Females (2** **m**.**o**.**)**	41	15

Only genes showing fold change >2 and Benjamini Hochberg adjusted P < 0.05 were considered as differentially expressed.

**Table 2:** Fold change of kidney gene expression in Tg^*Umod*C147W^ mice relative to age- and sex-matched Tg^*Umod*wt^.

Gene	Males (1 m.o.)		Females (1 m.o.)		Females (2 m.o.)	
	RT-qPCR	Microarray	RT-qPCR	Microarray	RT-qPCR	Microarray
***Col1a1***	1.79**	2.09^§§§^	1.73**	2.20^§§§^	2.65***	2.53^§§§^
***Ntn4***	1.59**	1.48^§§§^	1.17	1.32^§§^	1.52*	1.46^§§^
***Panx1***	2.30**	1.16^§^	2.07***	1.22^§^	2.16***	1.39^§§§^
***Slc13a3***	0.48*	0.67^§§§^	0.56**	0.68^§§^	0.56*	0.71^§§^
***Slc25a17***	0.47***	0.53^§§§^	0.34***	0.46^§§§^	0.35*	0.41^§§§^

*P < 0.05; **P < 0.01; ***P < 0.001 (unpaired *t*-test).

^§^ *P* < 0.05; ^§§^ *P* < 0.01; ^§§§^ *P* < 0.001 (Benjamini Hochberg adjusted).

### Pathways related to inflammation, fibrosis and lipid metabolism are altered in the kidneys of 1 month old Tg^*Umod*C147W^

We carried out pathway analysis on transcriptional data from 1 month-old transgenic mice by combining results from GSEA (Gene Set Enrichment Analysis) (FDR or *q* value < 0.05) and DAVID functional annotation tools.

**Figure 3:**
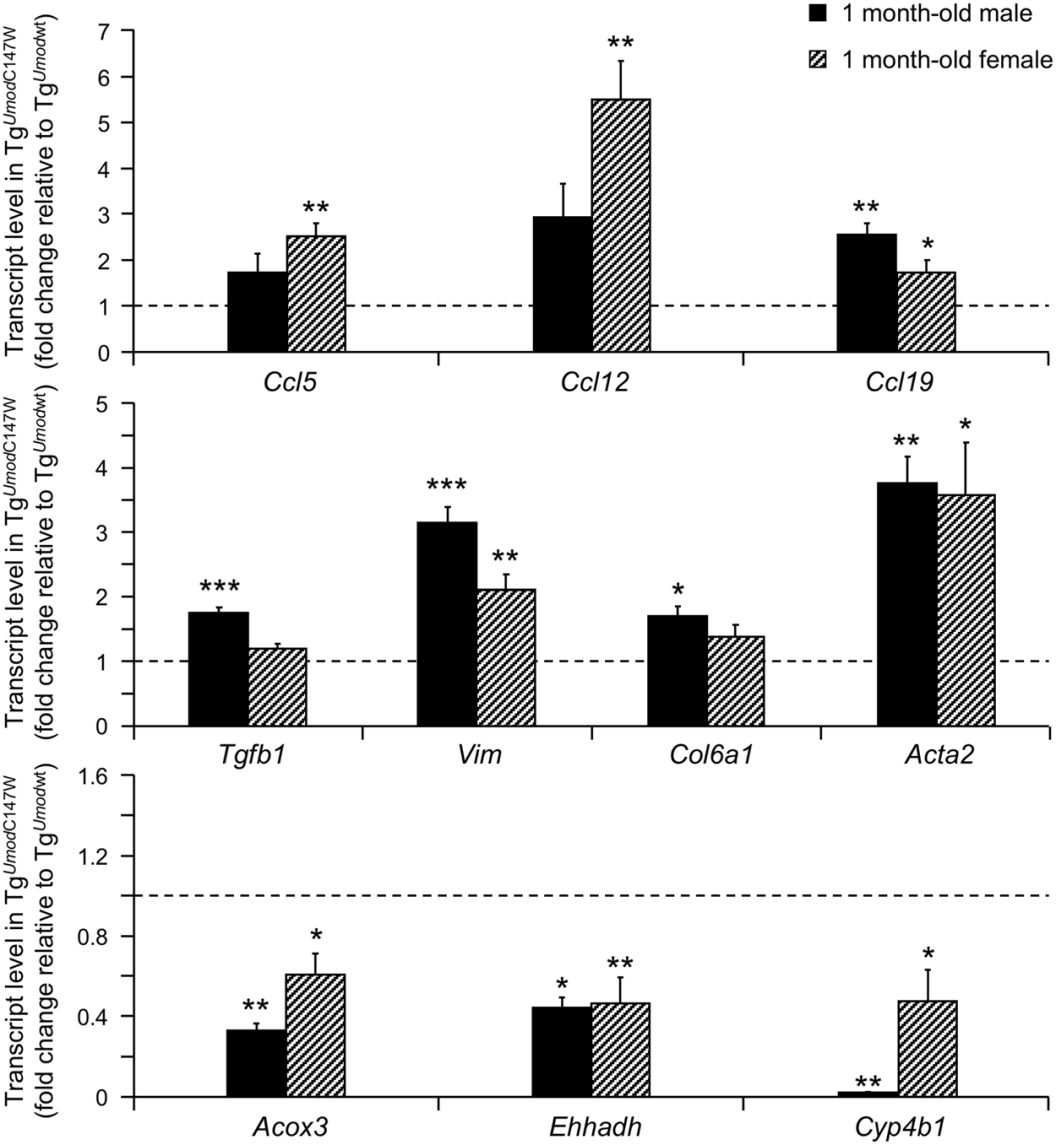
Renal transcript levels of genes belonging to inflammation (upper panel), fibrosis (middle panel) and lipid metabolism (lower panel) pathways, as detected by real-time RT-qPCR in 1 month-old male or female Tg^*Umod*C147W^ mice. Gene expression is reported as relative to age- and sex-matched Tg^*Umod*wt^ mice (n = 5/group). Data are expressed as mean ± s.e.m. *P < 0.05; **P < 0.01; ***P < 0.001 (unpaired *t*-test).

Clustering analysis with GSEA using Kyoto Encyclopedia of Genes and Genomes (KEGG) database shows up-regulation of pathways related to inflammation and fibrosis, and a decrease of fatty acid and amino-acid metabolism (Tables 3 and 4). Consistently, clustering of differentially expressed genes according to their cellular localization shows up-regulation of components of the extracellular matrix and down-regulation of peroxisomal and mitochondrial genes (data not shown). Very similar results were obtained performing analysis with DAVID (data not shown). Identification of fibrosis and inflammation as the most represented signatures in 1 month-old Tg^*Umod*C147W^ mice is interesting, as no clear signs of renal fibrosis or inflammation were detected at the histological level in these mice (Fig. 1). In order to confirm up-regulation of inflammatory pathways we assessed by RT-qPCR the expression of chemokines (i.e. *Ccl5*, *Ccl12* and *Ccl19*) that are up-regulated at later time points in Tg^*Umod*C147W^ mice (Supplementary Figure 3). Interestingly, transcripts of these chemokines are indeed already increased in the kidneys of mutant mice at 1 month of age (Fig. 3). Also, we confirmed by RT-qPCR the increased expression of genes involved in fibrosis (i.e. *Tgfb1*, *Vim*, *Col6a1* and *Acta2*) and the reduced expression of genes belonging to lipid metabolism pathways (i.e. *Acox3*, *Ehhadh* and *Cyp4b1*) (Fig. 3).

**Table 3:** List of the top 10 KEGG pathways up- or down-regulated in 1 month-old female Tg^*Umod*C147W^ mice relative to age- and sex-matched Tg^*Umod*wt^.

Up-regulated pathway	FDR	Number of genes	Contributing genes
ECM RECEPTOR INTERACTION	0	31/55 (84)	*Itgb1*, *Sdc3*, *Col1a2*, *Sdc1*, *Itga3*, *Lama5*, *Tnxb*, *Lamb2*, *Sv2a*, *Vwf*, *Sdc2*, *Lamc1*, *Itga11*, *Agrn*, *Lama2*, *Tnn*, *Col4a1*, *Hspg2*, *Col6a2*, *Thbs2*, *Col4a2*, *Col6a1*, *Fn1*, *Itgb4*, *Lamc2*, *Col6a3*, *Cd44*, *Col5a1*, *Col3a1*, *Tnc*, *Col1a1*
FOCAL ADHESION	0	51/133 (201)	*Pdgfa*, *Pdgfrb*, *Met*, *Itgb1*, *Pik3r3*, *Col1a2*, *Itga3*, *Lama5*, *Tln2*, *Birc2*, *Ppp1ca*, *Tnxb*, *Lamb2*, *Pdgfb*, *Vwf*, *Ptk2*, *Vegfc*, *Ilk*, *Cav2*, *Ppp1cb*, *Akt3*, *Shc1*, *Actn4*, *Lamc1*, *Itga11*, *Cav1*, *Figf*, *Lama2*, *Tnn*, *Col4a1*, *Actb*, *Vcl*, *Col6a2*, *Capn2*, *Thbs2*, *Col4a2*, *Col6a1*, *Mylk*, *Pdgfra*, *Fn1*, *Itgb4*, *Flnc*, *Lamc2*, *Flna*, *Col6a3*, *Col5a1*, *Actn1*, *Col3a1*, *Myl9*, *Tnc*, *Col1a1*
DNA REPLICATION	0	17/29 (36)	*Pola2*, *Rfc1*, *Rfc4*, *Pold2*, *Rfc3*, *Rfc5*, *Rpa1*, *Pold1*, *Rpa2*, *Mcm7*, *Fen1*, *Pole*, *Lig1*, *Mcm2*, *Mcm4*, *Mcm6*, *Mcm5*
REGULATION OF ACTIN CYTOSKELETON	0.001	60/133 (216)	*Actn2*, *Was*, *Fgfr2*, *Arhgef1*, *Nckap1*, *Arpc2*, *Rac1*, *Vav3*, *Rac2*, *Mapk1*, *Rock2*, *Fgfr1*, *Itga1*, *Arpc1a*, *Pfn2*, *Rac3*, *Abi2*, *Arpc5*, *Tmsb4x*, *Pdgfa*, *Pdgfrb*, *Limk1*, *Itgb1*, *Pik3r3*, *Pip5k1a*, *Pip4k2a*, *Itga3*, *Nras*, *Myh10*, *Ppp1ca*, *Wasf2*, *Pdgfb*, *Mras*, *Ptk2*, *Limk2*, *Pfn1*, *Tiam1*, *Ppp1cb*, *Fgf10*, *Actn4*, *Arhgef4*, *Itga11*, *Iqgap1*, *Rras*, *Nckap1l*, *Myh9*, *Arpc1b*, *Msn*, *Actb*, *Vcl*, *Scin*, *Gsn*, *Mylk*, *Pdgfra*, *Fn1*, *Itgb4*, *Actn1*, *Myl9*, *Cd14*, *F2r*
LEUKOCYTETRANSENDOTHELIAL MIGRATION	0.001	38/72 (118)	*Cldn23*, *Esam*, *Actn2*, *Ctnnb1*, *Mapk12*, *Rac1*, *Mapk11*, *Vav3*, *Rac2*, *Rock2*, *Cldn15*, *Cdh5*, *Gnai2*, *Mmp9*, *Ptpn11*, *Itgb1*, *Cldn7*, *Pik3r3*, *Pecam1*, *Cldn6*, *Ptk2*, *Ctnna1*, *Actn4*, *Ncf4*, *Mapk13*, *Msn*, *Actb*, *Vcl*, *Cldn19*, *Icam1*, *Cldn4*, *Cldn16*, *Cxcl12*, *Thy1*, *Mmp2*, *Actn1*, *Vcam1*, *Myl9*
GAP JUNCTION	0.001	19/36 (90)	*Adcy2*, *Tjp1*, *Mapk1*, *Adcy4*, *Plcb3*, *Gnai2*, *Gnas*, *Pdgfa*, *Pdgfrb*, *Csnk1d*, *Nras*, *Adcy1*, *Pdgfb*, *Prkx*, *Prkg1*, *Gja1*, *Tubb6*, *Gucy1a3*, *Itpr3*, *Htr2b*, *Tuba1b*, *Pdgfra*, *Tuba1a*, *Tubb2b*
PATHOGENIC ESCHERICHIA COLI INFECTION	0.001	19/36 (59)	*Was*, *Ctnnb1*, *Ncl*, *Arpc2*, *Rock2*, *Arpc1a*, *Cdh1*, *Tlr4*, *Arpc5*, *Itgb1*, *Hcls1*, *Arpc1b*, *Actb*, *Tubb6*, *Tuba1b*, *Tuba1a*, *Krt18*, *Cd14*, *Tubb2b*
CYTOKINE CYTOKINE RECEPTOR INTERACTION	0.002	28/81 (267)	*Cx3cr1*, *Pdgfa*, *Pdgfrb*, *Met*, *Il11*, *Tgfb3*, *Ifngr1*, *Pdgfb*, *Tnfrsf1a*, *Vegfc*, *Tgfbr2*, *Ccl7*, *Ccl4*, *Ccl19*, *Bmpr1b*, *Figf*, *Ccl5*, *Cxcl14*, *Il28ra*, *Tnfsf13b*, *Tnfrsf21*, *Cxcl1*, *Pdgfra*, *Cxcl12*, *Tnfrsf12a*, *Cx3cl1*, *Inhba*, *Osmr*
RIBOSOME	0.002	47/55 (88)	*Uba52*, *Rpl4*, *Rpl34*, *Rpl22*, *Rpl26*, *Rps29*, *Rpl19*, *Rpl18a*, *Rps27a*, *Rpl35*, *Rps10*, *Rps3a*, *Rps15*, *Rpl32*, *Rpl28*, *Rpl7a*, *Rps24*, *Rpl37*, *Rps19*, *Rps2*, *Rps21*, *Rpl9*, *Rpl13a*, *Rps7*, *Rpl27a*, *Rpl27*, *Rps5*, *Rpl10a*, *Rps15a*, *Rpl36al*, *Rpl24*, *Rplp0*, *Rpl30*, *Rps12*, *Rps13*, *Rps8*, *Rpl39*, *Rpl3*, *Rpl18*, *Rpl12*, *Rps9*, *Rps16*, *Rplp2*, *Rps3*, *Rpl36a*, *Rpl22l1*, *Rpl31*
CELL CYCLE	0.010	48/80 (128)	*Zbtb17*, *Cdkn1c*, *Anapc2*, *Mdm2*, *Cul1*, *Ccnd3*, *Ywhaz*, *Smad2*, *Cdc16*, *Stag1*, *Ywhag*, *Tgfb1*, *E2f3*, *Ywhab*, *Cdkn2d*, *Hdac2*, *Smc3*, *Smc1a*, *Gadd45g*, *Fzr1*, *Rbl1*, *Mad2l2*, *Anapc5*, *Ywhae*, *Skp2*, *Gadd45b*, *Tgfb3*, *E2f2*, *Smad3*, *Cdk4*, *Cdk2*, *Tfdp1*, *Dbf4*, *Plk1*, *Cdc20*, *Ccnb1*, *Mad2l1*, *Ywhah*, *Mcm7*, *Bub1b*, *Cdc6*, *Mcm2*, *Cdkn2b*, *Cdc7*, *Mcm4*, *E2f1*, *Mcm6*, *Mcm5*
**Down-regulated pathway**	**FDR**	**Number of genes**	**Contributing genes**
GLYCINE SERINE AND THREONINE METABOLISM	0	15/23 (31)	*Shmt1*, *Amt*, *Gatm*, *Gamt*, *Srr*, *Psat1*, *Shmt2*, *Gldc*, *Glyctk*, *Pipox*, *Agxt2*, *Sardh*, *Gnmt*, *Dmgdh*, *Cbs*
PEROXISOME	0	31/61 (78)	*Gnpat*, *Mlycd*, *Pex26*, *Nudt19*, *Pex11a*, *Mpv17*, *Acsl1*, *Dhrs4*, *Pex7*, *Pex3*, *Acot8*, *Pxmp2*, *Pex5*, *Pex6*, *Scp2*, *Pxmp4*, *Gstk1*, *Hmgcl*, *Acox3*, *Sod1*, *Cat*, *Ddo*, *Prdx5*, *Abcd4*, *Pipox*, *Pex1*, *Pecr*, *Ephx2*, *Hacl1*, *Ehhadh*, *Slc25a17*
BUTANOATE METABOLISM	0	17/21 (34)	*Aldh3a2*, *Bdh2*, *Acsm5*, *Hmgcs2*, *Aldh2*, *Oxct1*, *Aldh1b1*, *Acads*, *Acat1*, *Hmgcl*, *L2hgdh*, *Aldh5a1*, *Abat*, *Bdh1*, *Ehhadh*, *Aacs*, *Acsm3*
VALINE LEUCINE AND ISOLEUCINE DEGRADATION	0	19/33 (44)	*Hsd17b10*, *Aldh6a1*, *Aldh3a2*, *Mccc2*, *Bcat1*, *Hmgcs2*, *Pccb*, *Aldh2*, *Mccc1*, *Oxct1*, *Aldh1b1*, *Acads*, *Acat1*, *Hmgcl*, *Abat*, *Ivd*, *Bckdha*, *Acadm*, *Ehhadh*
PYRUVATE METABOLISM	0.006	11/25 (40)	*Aldh2*, *Hagh*, *Grhpr*, *Aldh1b1*, *Mdh1*, *Acat1*, *Acss2*, *Pck1*, *Pklr*, *Acot12*, *Ldhd*
TYROSINE METABOLISM	0.010	8/18 (42)	*Aldh3b1*, *Fah*, *Hgd*, *Hemk1*, *Nat6*, *Comt*, *Hpd*, *Ddc*
PROPANOATE METABOLISM	0.011	13/20 (33)	*Ehhadh*, *Acadm*, *Abat*, *Acss2*, *Acat1*, *Aldh1b1*, *Suclg2*, *Aldh2*, *Suclg1*, *Pccb*, *Mlycd*, *Aldh3a2*, *Aldh6a1*
PPAR SIGNALING PATHWAY	0.018	23/41 (69)	*Apoc3*, *Ehhadh*, *Lpl*, *Cyp27a1*, *Aqp7*, *Ppard*, *Acadm*, *Nr1h3*, *Acox3*, *Pck1*, *Ppara*, *Fads2*, *Scp2*, *Rxra*, *Slc27a1*, *Acsl1*, *Acadl*, *Hmgcs2*, *Adipoq*, *Pdpk1*, *Cpt2*, *Rxrb*, *Dbi*
DRUG METABOLISM CYTOCHROME P450	0.020	15/29 (72)	*Cyp2e1*, *Fmo4*, *Fmo1*, *Gstk1*, *Gsta3*, *Gstm1*, *Gstt1*, *Gsto2*, *Gstm5*, *Aldh3b1*, *Gstt2*, *Gstp1*, *Mgst3*, *Gsta2*, *Fmo5*
SELENOAMINO ACID METABOLISM	0.038	5/20 (26)	*Cbs*, *Ahcy*, *Hemk1*, *Scly*, *Papss2*

FDR, False Discovery Rate. Number of genes, A/B (C): A, number of genes contributing to pathway enrichment; B, number of genes from the KEGG pathway expressed in analysed experimental groups; C, total number of genes listed in the KEGG pathway.

**Table 4:** List of the top 10 KEGG pathways up- and down-regulated in 1 month-old male Tg^*Umod*C147W^ mice relative to age- and sex-matched Tg^*Umod*wt^.

Up-regulated pathway	FDR	Number of genes	Contributing genes
FOCAL ADHESION	0	58/138 (201)	*Pik3r1*, *Ptk2*, *Prkca*, *Crk*, *Col1a2*, *Ppp1r12a*, *Itga11*, *Zyx*, *Ilk*, *Tnxb*, *Igf1*, *Vwf*, *Itga10*, *Itga3*, *Vav3*, *Itga9*, *Egf*, *Itga5*, *Vcl*, *Flnc*, *Akt3*, *Shc1*, *Capn2*, *Actn4*, *Itgb6*, *Vegfc*, *Tnn*, *Jun*, *Col4a2*, *Pdgfrb*, *Actb*, *Ccnd2*, *Col4a1*, *Pdgfb*, *Lamb2*, *Thbs1*, *Lamc1*, *Flna*, *Itgb4*, *Cav1*, *Lamc2*, *Mylk*, *Figf*, *Actn1*, *Pik3r3*, *Lama2*, *Thbs2*, *Spp1*, *Col3a1*, *Col6a3*, *Col6a2*, *Col6a1*, *Pdgfra*, *Col5a1*, *Fn1*, *Col1a1*, *Tnc*, *Myl9*
ECM RECEPTOR INTERACTION	0	34/57 (84)	*Vtn*, *Col1a2*, *Cd47*, *Itga11*, *Sv2a*, *Tnxb*, *Vwf*, *Itga10*, *Itga3*, *Itga9*, *Agrn*, *Itga5*, *Itgb6*, *Tnn*, *Col4a2*, *Cd44*, *Col4a1*, *Lamb2*, *Thbs1*, *Hspg2*, *Lamc1*, *Itgb4*, *Lamc2*, *Lama2*, *Thbs2*, *Spp1*, *Col3a1*, *Col6a3*, *Col6a2*, *Col6a1*, *Col5a1*, *Fn1*, *Col1a1*, *Tnc*
RIBOSOME	0.001	37/52 (88)	*Rpl39*, *Rpl23*, *Rps19*, *Rpl36a*, *Rps21*, *Rpl9*, *Rpl3*, *Rpl27a*, *Rpl19*, *Rps11*, *Rpl32*, *Rplp2*, *Rps5*, *Rps2*, *Rps10*, *Rpl38*, *Rpl7a*, *Rpl22*, *Rpl28*, *Rpl35*, *Rps15a*, *Rpl30*, *Rpl37*, *Rps12*, *Rps15*, *Rps8*, *Rpl18*, *Rpl18a*, *Rpl34*, *Rpl26*, *Rps6*, *Rps26*, *Rps24*, *Rps9*, *Rpl31*, *Rps3*, *Rps16*
DILATED CARDIOMYOPATHY	0.001	22/48 (92)	*Prkx*, *Tgfb3*, *Itga11*, *Tgfb1*, *Igf1*, *Itga10*, *Itga3*, *Itga9*, *Gnas*, *Atp2a2*, *Itga5*, *Itgb6*, *Actb*, *Adcy4*, *Cacnb3*, *Adcy6*, *Itgb4*, *Slc8a1*, *Tpm2*, *Tpm4*, *Lama2*, *Tpm1*
GAP JUNCTION	0.002	23/51 (90)	*Prkca*, *Nras*, *Prkx*, *Csnk1d*, *Gja1*, *Gnai2*, *Tuba1b*, *Itpr2*, *Egf*, *Gnas*, *Gucy1b3*, *Prkg1*, *Pdgfrb*, *Tubb6*, *Adcy4*, *Pdgfb*, *Adcy6*, *Itpr3*, *Htr2b*, *Gucy1a3*, *Tuba1a*, *Pdgfra*, *Tubb2b*
LEUKOCYTE TRANSENDOTHELIAL MIGRATION	0.002	31/73 (118)	*Ptpn11*, *Mapk12*, *Ctnna1*, *Pik3r1*, *Ptk2*, *Prkca*, *Icam1*, *Cxcl12*, *Rassf5*, *Cxcr4*, *Cldn15*, *Pecam1*, *Gnai2*, *Vav3*, *Plcg2*, *Msn*, *Esam*, *Vcl*, *Cdh5*, *Actn4*, *Actb*, *Cldn19*, *Thy1*, *Actn1*, *Pik3r3*, *Cldn16*, *Cldn4*, *Vcam1*, *Cldn10*, *Mmp2*, *Myl9*
ARRHYTHMOGENIC RIGHT VENTRICULAR CARDIOMYOPATHY ARVC	0.009	24/40 (76)	*Itgb1*, *Dag1*, *Emd*, *Itgb5*, *Cacna2d1*, *Jup*, *Ctnna1*, *Itga11*, *Gja1*, *Itga10*, *Itga3*, *Itga9*, *Atp2a2*, *Itga5*, *Actn4*, *Itgb6*, *Dsp*, *Actb*, *Cacnb3*, *Itgb4*, *Slc8a1*, *Actn1*, *Lama2*, *Dsg2*
HYPERTROPHIC CARDIOMYOPATHY HCM	0.015	18/45 (85)	*Tgfb3*, *Itga11*, *Tgfb1*, *Igf1*, *Itga10*, *Itga3*, *Itga9*, *Atp2a2*, *Itga5*, *Itgb6*, *Actb*, *Cacnb3*, *Itgb4*, *Slc8a1*, *Tpm2*, *Tpm4*, *Lama2*, *Tpm1*
VASCULAR SMOOTH MUSCLE CONTRACTION	0.019	24/63 (115)	*Pla2g12a*, *Prkca*, *Ppp1r12a*, *Prkx*, *Pla2g4b*, *Itpr2*, *Gnas*, *Gucy1b3*, *Mrvi1*, *Myh11*, *Actg2*, *Ramp2*, *Prkg1*, *Ednra*, *Myl6*, *Adcy4*, *Adcy6*, *Itpr3*, *Mylk*, *Avpr1a*, *Adora2b*, *Gucy1a3*, *Myl9*, *Acta2*
CARDIAC MUSCLE CONTRACTION	0.019	20/35 (80)	*Cox6c*, *Cox6b1*, *Cox7b*, *Slc9a1*, *Tpm3*, *Atp1a2*, *Cacna2d1*, *Atp1b3*, *Cox7a2l*, *Cyc1*, *Cox7a1*, *Atp2a2*, *Cox6b2*, *Cacnb3*, *Atp1b2*, *Slc8a1*, *Tpm2*, *Fxyd2*, *Tpm4*, *Tpm1*
**Down-regulated pathway**	**FDR**	**Number of genes**	**Contributing genes**
PEROXISOME	0.001	36/63 (78)	*Nudt19*, *Ephx2*, *Pecr*, *Ehhadh*, *Slc25a17*, *Acox3*, *Pipox*, *Nudt12*, *Far1*, *Agps*, *Cat*, *Acox1*, *Mlycd*, *Prdx5*, *Ddo*, *Abcd3*, *Amacr*, *Scp2*, *Dhrs4*, *Gnpat*, *Pxmp4*, *Slc27a2*, *Sod1*, *Pex3*, *Pex16*, *Gstk1*, *Pex2*, *Pex7*, *Abcd4*, *Pex14*, *Hmgcl*, *Crat*, *Pex11a*, *Prdx1*, *Mpv17*, *Sod2*
BUTANOATE METABOLISM	0.001	11/21 (34)	*Acsm3*, *Aacs*, *Acat1*, *Ehhadh*, *Bdh1*, *Aldh3a2*, *Acsm5*, *Aldh5a1*, *L2hgdh*, *Acads*, *Abat*
PROPANOATE METABOLISM	0.015	8/19 (33)	*Acat1*, *Ehhadh*, *Mlycd*, *Aldh3a2*, *Suclg2*, *Abat*, *Acss2*, *Aldh6a1*
PYRUVATE METABOLISM	0.019	10/26 (40)	*Ldhd*, *Acot12*, *Acat1*, *Pck1*, *Aldh3a2*, *Acss2*, *Mdh1*, *Grhpr*, *Pklr*, *Dld*
GLYCINE SERINE AND THREONINE METABOLISM	0.019	11/24 (31)	*Cbs*, *Pipox*, *Shmt2*, *Gnmt*, *Sardh*, *Srr*, *Chdh*, *Dmgdh*, *Glyctk*, *Agxt2*, *Gamt*
CYSTEINE AND METHIONINE METABOLISM	0.020	8/19 (34)	*Cbs*, *Ahcy*, *Mat2a*, *Mpst*, *Ahcyl1*, *Mtr*, *Trdmt1*, *Mat2b*
SELENOAMINO_ACID_METABOLISM	0.020	9/21 (26)	*Cbs*, *Ahcy*, *Ggt1*, *Mat2a*, *Ahcyl1*, *Papss1*, *Mat2b*, *Mars2*, *Sephs2*
TRYPTOPHAN METABOLISM	0.065	9/23 (40)	*Inmt*, *Acat1*, *Haao*, *Ehhadh*, *Afmid*, *Cat*, *Aldh3a2*, *Gcdh*, *Kmo*
LYSINE DEGRADATION	0.069	8/34 (64)	*Acat1*, *Aass*, *Ehhadh*, *Pipox*, *Plod2*, *Aldh3a2*, *Plod3*, *Gcdh*
SYSTEMIC LUPUS ERYTHEMATOSUS	0.071	19/56 (140)	*C8a*, *C8g*, *Hist1h4h*, *Hist1h4i*, *Hist1h4a*, *Hist1h4f*, *Hist1h4j*, *Hist1h2be*, *Hist1h2bn*, *Hist1h2bk*, *Hist1h4k*, *Hist1h2bj*, *Hist1h2bm*, *Hist1h2bc*, *Hist1h2bh*, *Fcgr2b*, *Hist1h2bf*, *C4b*, *C4a*

FDR, False Discovery Rate. Number of genes, A/B (C): A, number of genes contributing to pathway enrichment; B, number of genes from the KEGG pathway expressed in analysed experimental groups; C, total number of genes listed in the KEGG pathway.

**Figure 4:**
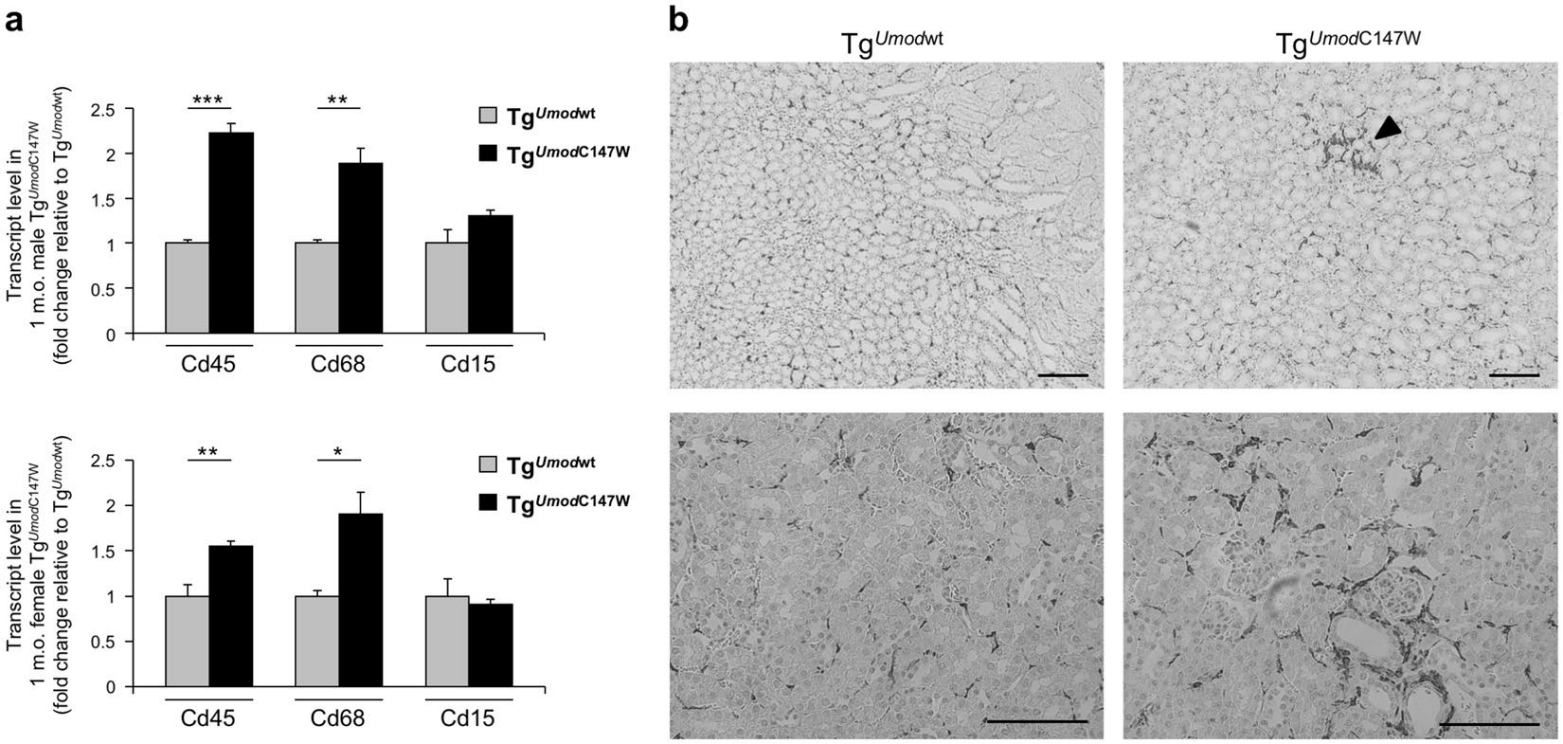
Characterization of infiltrating cells in kidneys of Tg^*Umod*C147W^ mice at 1 month of age. (**a**) Analysis by RT-qPCR of the expression of markers of infiltrating cells in male (upper panel) or female (bottom panel) Tg^*Umod*C147W^ mice relative to age- and sex-matched Tg^*Umod*wt^ mice (n = 5/group). *Cd45* (*Ptprc*, common leukocyte marker), *Cd68* (macrophage), *Cd15* (*Fut4*, granulocyte) transcripts were highly expressed, while *Cd5* (T cells) and *Cd19* (B cells) were barely detectable (data not shown). Data are expressed as mean ± s.e.m. *P < 0.05; **P < 0.01; ***P < 0.001 (unpaired *t*-test). (**b**) Representative immunohistochemistry (IHC) staining for macrophage marker F4/80 in kidneys of Tg^*Umod*C147W^ and Tg^*Umod*wt^ mice. IHC analysis shows focal increase (arrowhead) of macrophage infiltrate in Tg^*Umod*C147W^ mice (scale bar 100 µm).

We further characterised inflammation in the kidneys of 1 month-old Tg^*Umod*C147W^ mice by assessing the expression of markers of inflammatory cells by RT-qPCR analysis. While expression of *Cd5* (T cells) and *Cd19* (B cells) was barely detectable (Cp > 35), we found robust expression of *Cd45* (*Ptprc*) (common leukocyte marker), *Cd68* (macrophage) and *Cd15* (*Fut4)* (granulocyte). Relative expression analysis indicates increased infiltrating inflammatory cells, essentially macrophages, in the kidneys of Tg^*Umod*C147W^ mice (Fig. 4a). This was confirmed by immunohistochemistry analysis showing areas of focal macrophage invasion in the kidneys of 1 month-old Tg^*Umod*C147W^ mice (Fig. 4b).

### Inflammatory pathways are associated with renal disease progression in Tg^*Umod*C147W^ mice

**Figure 5:**
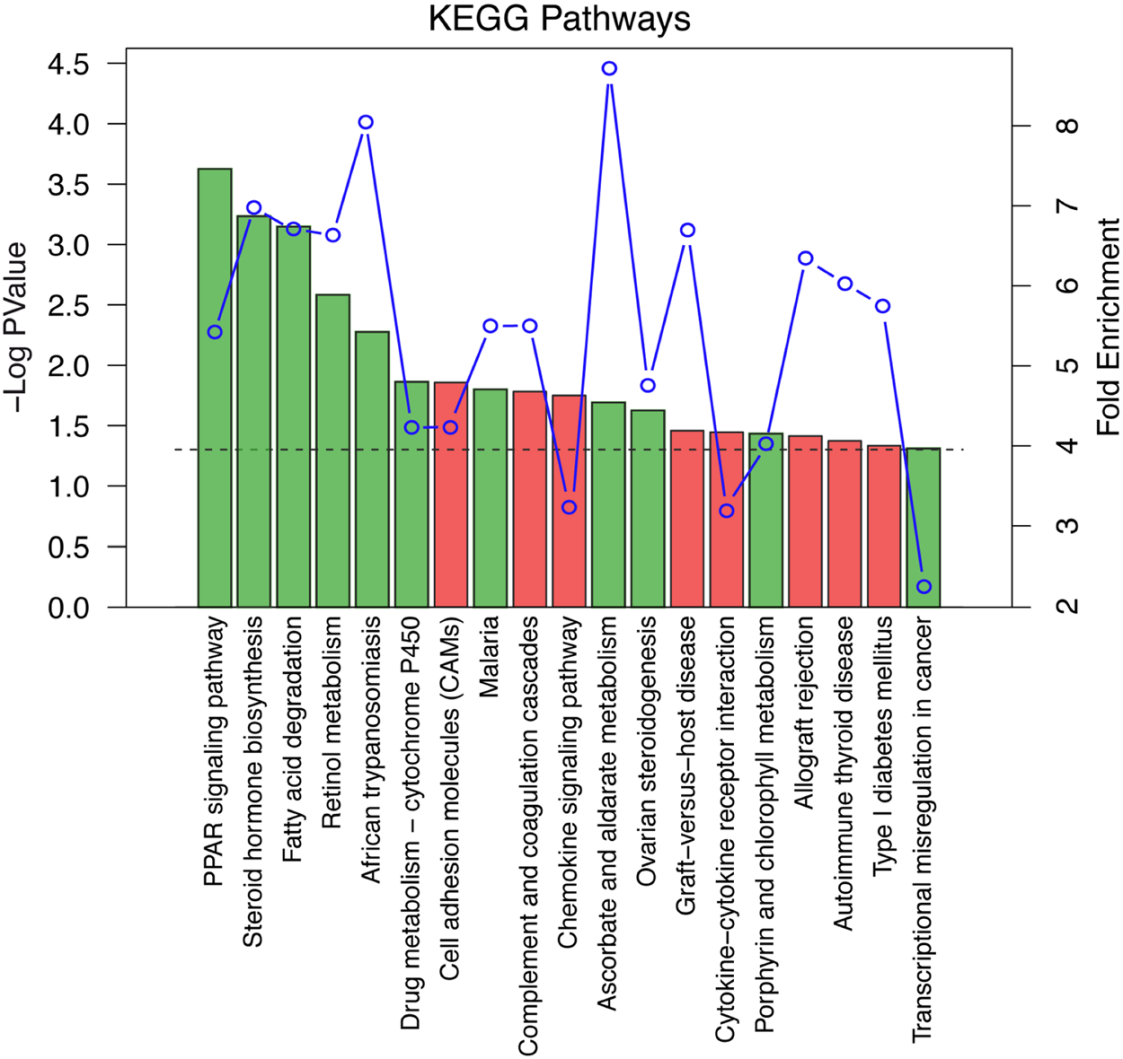
KEGG pathways enriched during the progression of renal disease in Tg^*Umod*C147W^ identified by clusterProfiler Bioconductor package. The analysed list is composed of genes that are significantly up- or down-regulated in Tg^*Umod*C147W^ female mice at 2 months of age, regardless of their fold change, and that are not differentially expressed at 1 month of age in sex-matched mice. Pathways up-regulated are represented by red bars while the down-regulated are green. The height of bars is proportional to the statistical significance of the term (dotted line corresponds to enrichment *P* value = 0.05). Fold enrichment of the significant terms is represented as a blue line. Fold enrichment is defined as the ratio gene frequency/background frequency (see Methods for details).

Pathway analysis on data from kidneys of 2 month-old mice confirmed findings on 1 month-old mice, with enrichment of genes related to inflammation and fibrosis (up-regulated) and to metabolism of fatty acids and amino acids (down-regulated) (data not shown). In order to identify pathways associated with disease progression, we compared transcriptional profiles derived from transgenic female mice at 1 and 2 months of age. We identified 477 genes that are significantly up- (196) or down-regulated (281) in Tg^*Umod*C147W^ mice at 2 months of age, regardless of their fold change, and that were not differentially expressed at 1 month of age. Pathway enrichment analysis (clusterProfiler Bioconductor package, see Methods) identified up-regulated pathways related to inflammation and immune response, highlighting their growing activation along with disease progression. Noteworthy, pathways related to fatty acid and lipid metabolism were significantly enriched among down-regulated ones (Fig. 5). We also identified few genes showing opposite behaviour in 1 and 2 month-old mice: 5 genes that resulted significantly up-regulated in 1 month-old (*A630097A12Rik*, *Adnp*, *Ren1*, *Spin*, *Ugt1a13*) and significantly down-regulated in 2 month-old Tg^*Umod*C147W^ mice and 5 genes (*4931408A02Rik*, *Gpx4*, *Igh-VJ558*, *LOC277193*, *Slc9a8*) that showed opposite direction of age-related change. Future work will be needed to establish the significance of these findings for disease progression.

### TAL stress and inflammatory signals represent an early event in ADTKD-*UMOD*

**Figure 6:**
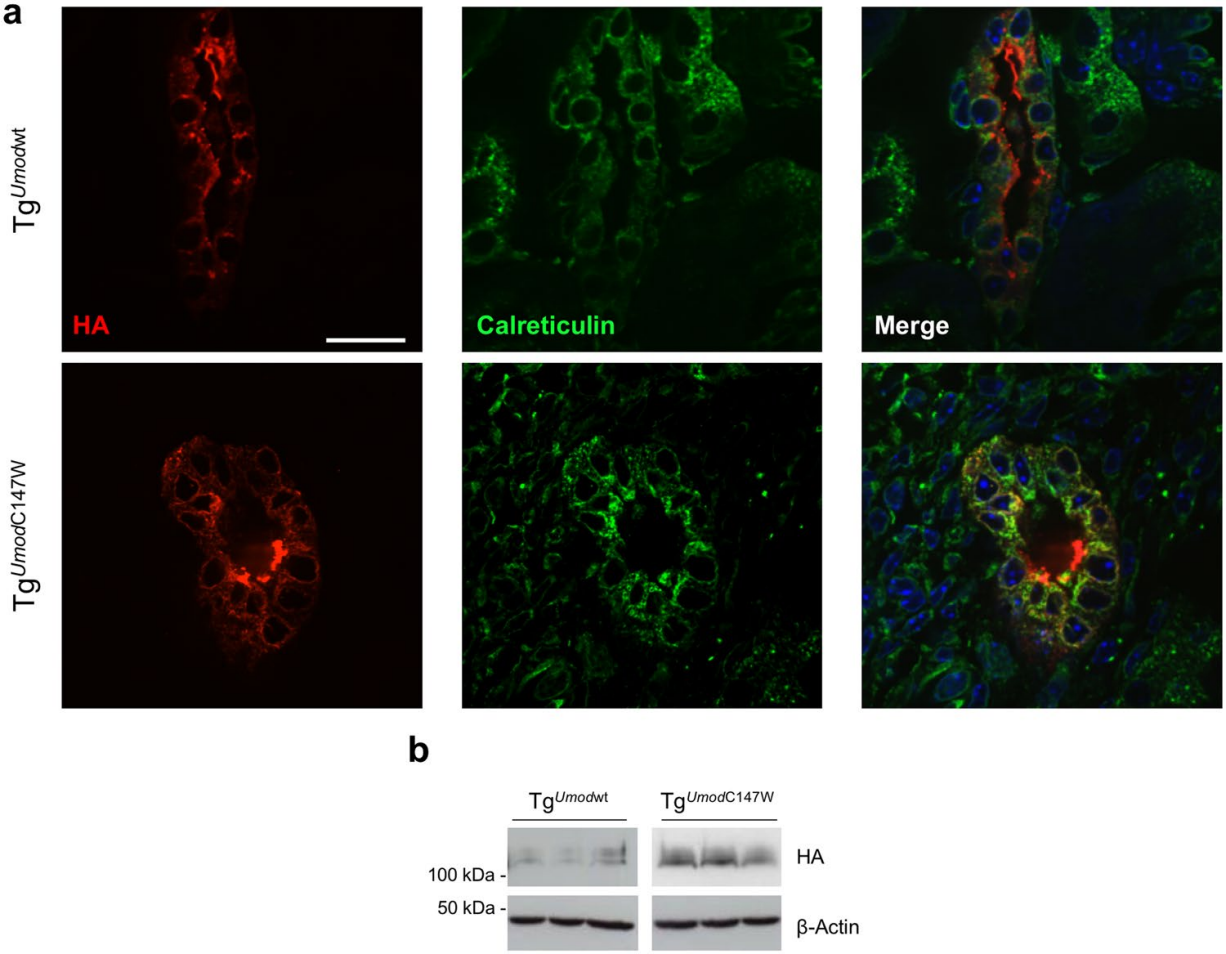
ER retention of mutant uromodulin in young Tg^*Umod*C147W^ mice. (**a**) Immunofluorescence analysis for transgenic uromodulin (HA) and the ER marker calreticulin in Tg^*Umod*wt^ (upper panel) and Tg^*Umod*C147W^ (lower panel) mice at p8. Wild-type transgenic uromodulin is enriched at the apical plasma membrane of TAL cells, while most of the transgenic mutant protein is retained in the ER (scale bar 15 µm). (**b**) ER retention of mutant uromodulin is also evident in Western blot experiment, in which samples from Tg^*Umod*C147W^ kidneys show a more intense signal for the lower molecular weight uromodulin isoform, corresponding to the protein ER precursor. The figure shows cropped blots (full blots are reported in Supplementary Figure 6).

**Figure 7:**
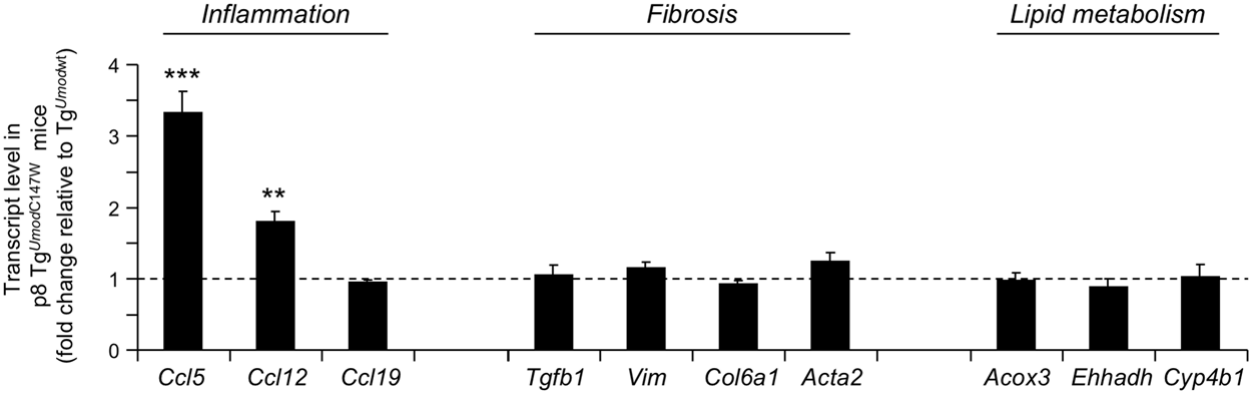
Expression level detected by RT-qPCR of genes involved in pathways of inflammation, fibrosis and lipid metabolism in kidneys of Tg^*Umod*C147W^ mice at p8 relative to age- and sex-matched Tg^*Umod*wt^ mice (n = 8 Tg^*Umod*wt^ and 6 Tg^*Umod*C147W^). Data are expressed as mean ± s.e.m. **P < 0.01; ***P < 0.001 (unpaired *t*-test).

**Figure 8:**
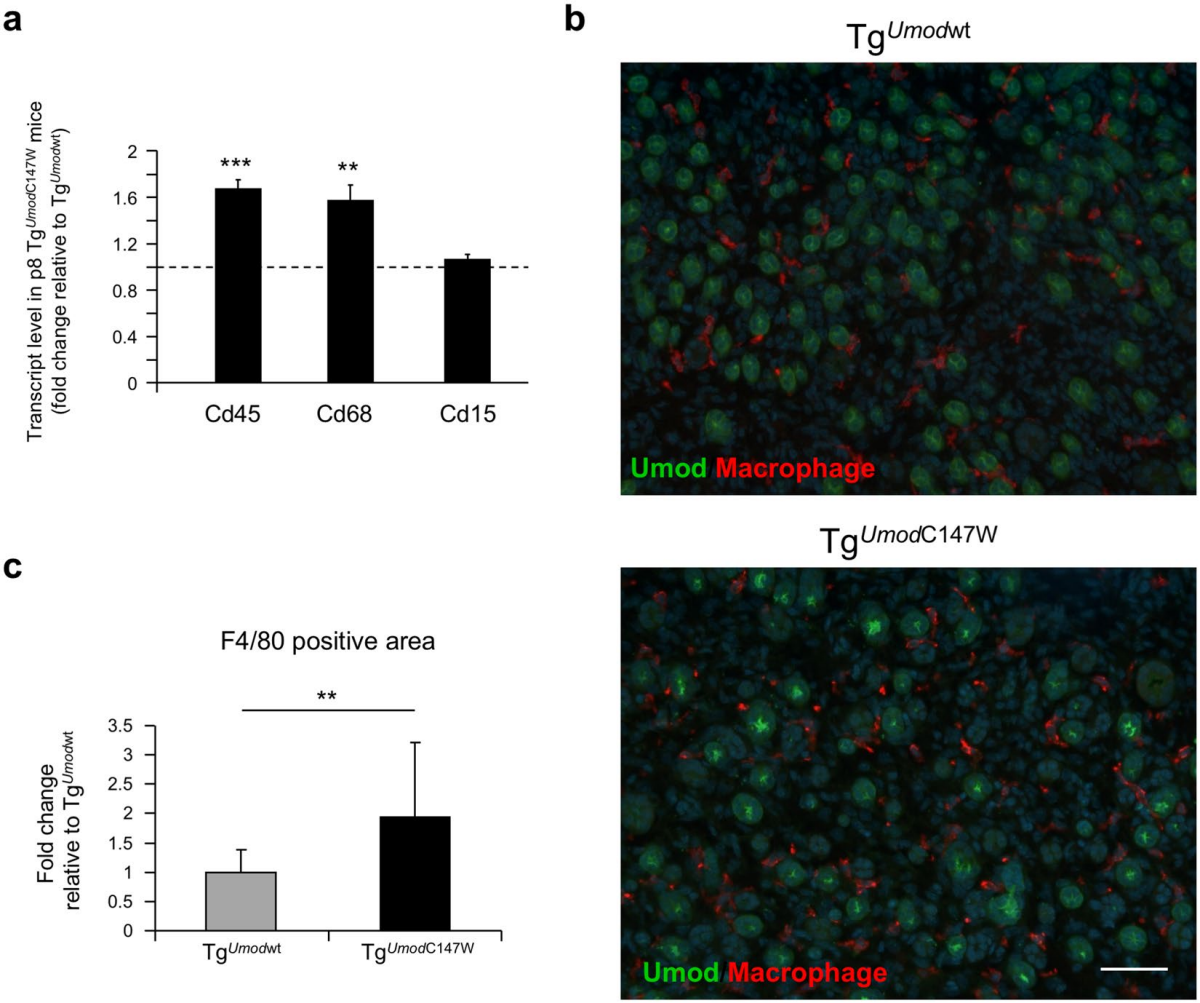
Inflammatory cell infiltrate in the kidneys of p8 Tg^*Umod*C147W^ mice. (**a**) Analysis by RT-qPCR of markers of infiltrating cells in Tg^*Umod*C147W^ mice relative to age- and sex-matched Tg^*Umod*wt^ mice (n = 8 Tg^*Umod*wt^ and 6 Tg^*Umod*C147W^). As in mice at 1 month of age, *Cd5* (T cells) and *Cd19* (B cells) are barely detectable (data not shown). Inflammatory infiltrating cells in the kidneys of Tg^*Umod*C147W^ mice are essentially represented by macrophages (*Cd68*). Data are expressed as mean ± s.e.m. **P < 0.01; ***P < 0.001 (unpaired *t*-test). (**b**) Representative images of immunofluorescence staining for macrophage marker (F4/80) in kidneys from Tg^*Umod*wt^ and Tg^*Umod*C147W^ mice at p8 (scale bar 50 µm). (**c**) Quantification of F4/80 positive area. Data are expressed as mean ± s.d. (n = 4 Tg^*Umod*wt^ and 5 Tg^*Umod*C147W^). **P < 0.01 (unpaired *t*-test).

Our data showed induction of pathways related to inflammation and fibrosis in the kidneys of Tg^*Umod*C147W^ mice already at 1 month of age, demonstrating that expression of mutant uromodulin induces release of pro-inflammatory and pro-fibrotic signals well before the presence of any sign of renal damage at the histological level. To gain further insight into the potential role of inflammatory/fibrotic signals in the disease onset, we analysed kidneys of mice 8 days *postpartum* (p8). Interestingly, ER retention of mutant uromodulin is already evident at this early time point (Fig. 6). In line with what observed at 1 month of age, evaluation of histological parameters reveals that kidneys of Tg^*Umod*C147W^ mice at p8 are similar to the ones of Tg^*Umod*wt^ mice (Supplementary Figure 4). We then analysed the transcript level of cytokines/chemokines, genes related to fibrosis and inflammatory cell markers in these kidneys. Interestingly, *Ccl5* and *Ccl12* genes were significantly increased in Tg^*Umod*C147W^ mice, while no differences were detected in expression of *Ccl19* and of transcripts related to fibrosis or lipid metabolism (Fig. 7). Upregulation of chemokines in p8 kidneys was associated with higher expression of markers of inflammatory cells, indicating increased macrophage infiltrate (Fig. 8a–c). These results suggest that the induction of pro-inflammatory signals is likely one of the first events occurring in the kidneys of Tg^*Umod*C147W^ mice following mutant uromodulin expression.

**Figure 9:**
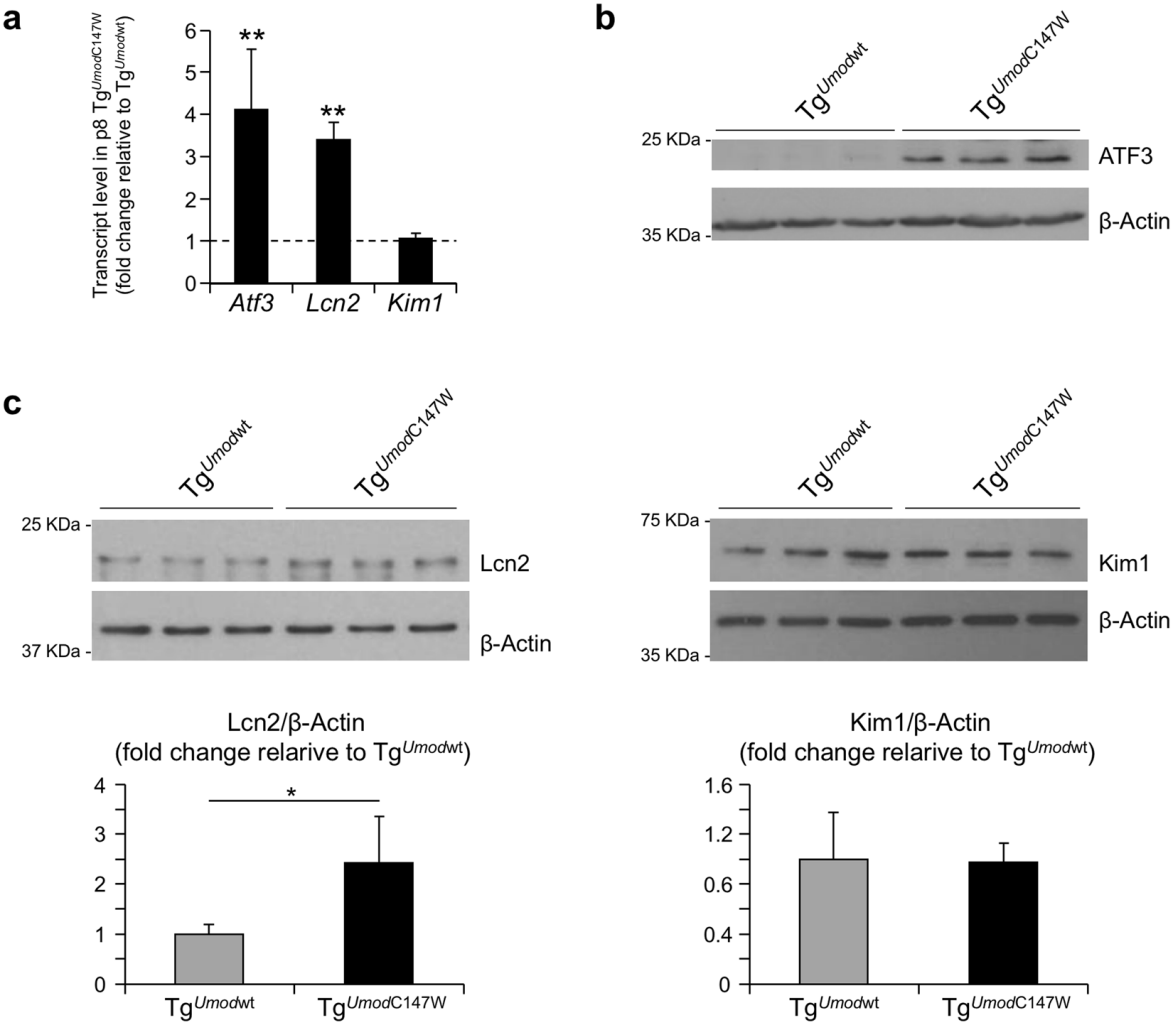
Expression of tubular damage and cell stress markers in the kidneys of p8 transgenic mice. (**a**) Expression level (RT-qPCR) of markers of distal tubular damage (*Lcn2*), of proximal tubular damage (*Kim1*) and of cellular stress (*Atf3*) in Tg^*Umod*C147W^ mice relative to age- and sex-matched Tg^*Umod*wt^ mice (n = 8 Tg^*Umod*wt^ and 6 Tg^*Umod*C147W^). Data are expressed as mean ± s.e.m. **P < 0.01 (unpaired *t*-test). (**b**) Western blot analysis showing increased expression of renal Atf3 protein in Tg^*Umod*C147W^ mice (n = 3/group). (**c**) Representative Western blot analysis of kidney lysates from Tg^*Umod*wt^ and Tg^*Umod*C147W^ mice showing expression of Lcn2 (n = 4 Tg^*Umod*wt^ and 5 Tg^*Umod*C147W^) and Kim1 (n = 3/group) in Tg^*Umod*wt^ and Tg^*Umod*C147W^ mice (upper panels), and relative quantification (bottom panels). Actin was used as a loading control. The figure shows cropped images (full blots are reported in Supplementary Figure 6). Data are expressed as mean ± s.d. *P < 0.05 (unpaired t-test).

We also checked the expression of *Lcn2* (lipocalin 2 or Neutrophil gelatinase-associated lipocalin (NGAL)) and *Hacvr1* (Hepatitis A virus receptor or Kidney injury molecule-1 (Kim1)) genes. *Lcn2* and *Havcr1* are well-established markers of distal and proximal tubule damage respectively that were found to be significantly up-regulated already at 1 month of age in the transcriptome analysis of Tg^*Umod*C147W^ kidneys. At p8 we detected both at transcript and protein level significant up-regulation of *Lcn2* while expression of *Kim1* was not different in Tg^*Umod*C147W^ mice (Fig. 9a,c). These results suggest that renal damage occurs in the distal tubules first, where uromodulin is expressed and is then spread to neighbouring proximal tubules. Data from microarray analysis show up-regulation of *Atf3* transcript. ATF3 is a transcription repressor that was shown to play a protective role in the kidney after ischemia/reperfusion injury through anti-inflammatory and anti-apoptotic effects in renal tubular epithelial cells^31^. It can be induced by a large variety of stresses, including ER stress, cytokine, genotoxic and cytotoxic agents^32^. Interestingly, we found increased expression of *Atf3* at transcript and protein level (Fig. 9a,b), specifically in TAL nuclei at p8 (Supplementary Figure 5 **)**. These data suggest early induction of cellular stress in TAL cells of Tg^*Umod*C147W^ mice.

## Discussion

In this study, we aimed at identifying early renal pathways triggered by expression of mutant uromodulin in a mouse model of ADTKD-*UMOD*. Our results derived from transcriptional profiling of kidneys from young adult mice show that inflammation and fibrosis are two of the most up-regulated pathways, while down-regulated ones are mostly related to lipid and amino acid metabolism. In line with this, clustering of DEGs according to their localization indicates up-regulation of components of the extracellular matrix and down-regulation of peroxisomal and mitochondrial genes. Moreover, the comparison of transcriptional data from 1 and 2 month-old Tg^*Umod*C147W^ mice indicate that inflammatory pathways may play a role in the progression of the disease.

Deregulation of some of the identified pathways has already been observed in other models of renal damage. By using a genetic model of nephronophthisis, i.e. mice lacking *Glis2*, it was shown that tubulointerstitial infiltrating cells and fibrosis are already present in kidneys of young animals^33^. Also, in a genetic model of Alport syndrome tubulointerstitial nephritis associated with presence of inflammatory cells is one of the major histological features^34^. Fibrotic pathways have also been shown to be up-regulated at 3 weeks of age in a rat model of polycystic kidney disease^35^. More recently, down-regulation of amino-acid and lipid metabolism has been associated with renal damage progression in humans and in mouse models of tubulointerstitial fibrosis^36^. Markers of lipid metabolism were indeed strongly reduced in fibrotic human kidneys. Moreover, restoring fatty acid metabolism by genetic or pharmacological methods protected mice from tubulointerstitial fibrosis^36^. Interestingly, also reduction of mitochondrial activity has been linked to kidney diseases^37^. Moreover, decreased number of functional peroxisomes was shown to worsen tubulointerstitial damage^38^. Overall, these data suggest that the main pathways that are dysregulated in kidneys of Tg^*Umod*C147W^ mice may reflect common features of chronic kidney disease onset and progression.

By using two different mouse lines carrying *Umod* mutations (*Umod* ^C93F^, *Umod* ^A227T^) induced by N-ethyl-N-nitrosourea (ENU), Kemter *et al*. showed that inflammation could play a role in models of ADTKD-*UMOD*, via activation of NF-kB pathway in TAL segments^39^. Consistently, pathway analysis of transcriptome data from female Tg^*Umod*C147W^ mice shows up-regulation of NF-kB pathway (Biocarta database, data not shown), suggesting that this pathway has a role also in Tg^*Umod*C147W^ mice. Moreover, Horsch *et al*. performed transcriptional profiling of kidneys from young-adult *Umod* ^A227T^ mice (17 weeks, mild disease model) and aged *Umod* ^C93F^ mice (38 weeks, advanced disease model). Overall, their analysis led to the identification of 104 and 54 DEGs at 17 and 38 weeks respectively, associated with cancer, cell proliferation, necrosis, inflammation, lipid and protein metabolism. About 15–30% of DEGs are also differentially expressed in Tg^*Umod*C147W^ mice. Discrepancies in the number/type of DEGs may be due to several differences, as genetic model (ENU mutant *versus* transgenic), *Umod* mutations, mouse strain, microarray platform (in-house produced versus commercially available, covering 17,346 genes and 48,804 transcripts respectively). Despite such differences, it is interesting to note that some of the main pathways identified in our study, as inflammation and lipid metabolism, were also detected in the study by Horsch *et al*. It is also of note that, similarly to what we observed, also in ENU models the number of DEGs decreases with disease progression.

Our data suggest that inflammation has relevance in the onset and progression of the renal disease in Tg^*Umod*C147W^ mice as we identified increased levels of chemokines in kidneys of mutant mice already at p8. At this time point, we were also able to find presence of infiltrating cells. Real time analyses showed that inflammatory cells are mainly represented by cells of the innate immunity type (i.e. macrophages) instead of cells of the adaptive immunity (i.e. T and B cells). This is in line with what observed in an Alport syndrome mouse model, that develops a chronic renal disease^34^. Although partial, our data also indicate that inflammation in *Umod* mutant mice precedes development of fibrosis. Indeed, transcript levels of *Tgfb*, a key factor regulating the fibrotic process that is found up-regulated in virtually any type of CKD in human and mouse models^40^, *Vim*, *Col6a1* and *Acta2* are identical in mutant and wild type transgenic mice at one week of age. Similarly to Tg^*Umod*C147W^ mice, inflammation preceding fibrosis was also described in several mouse models of induced renal damage^41^. Overall, these data suggest that inflammation could be a target of future pharmacological intervention directed at treatment of the pathology.

Early development of inflammation is coupled with tubular damage, as demonstrated by increased expression of *Lcn2* in kidneys from Tg^*Umod*C147W^ mice at p8. Lipocalin-2 transcript remains at high levels in adult mice, and it results as one of the most up-regulated DEGs in our transcriptome analysis. Lipocalin-2 is an established marker of distal tubule damage^42^ that regulates progression of CKD and cyst formation^43^. It was found to be highly up-regulated in renal tissues of other genetic models of progressive kidney damage^44^. *Havcr1*, a marker of proximal tubular damage^45^, is found to be up-regulated in adult Tg^*Umod*C147W^ mice used for the transcriptional analysis while is identical to Tg^*Umod*wt^ mice at p8. These results might indicate that tubular damage takes place in distal tubules, where uromodulin is expressed and then propagates to proximal tubules, possibly via a cross-talk between TAL and proximal tubular segments.

Starting from 8 days after birth, we also identified in kidneys of Tg^*Umod*C147W^ mice up-regulation of transcriptional repressor *Atf3* that remains at high levels in adult animals. ATF3 belongs to the activating transcription factor/cAMP responsive element-binding protein (ATF/CREB) family. It can be activated by several stimuli, including ER stress and unfolded protein response (UPR), chemokines and cytokines, genotoxic and cytotoxic agents. ATF3 plays a protective role after renal ischemic injury^31, 46, 47^ through anti-inflammatory and anti-apoptotic effects on epithelial cells^46^. The anti-inflammatory effect of ATF3 is thought to be exerted via chromatin remodelling and subsequent transcriptional inhibition of gene targets of NF-kB pathway^46, 48^. In Tg^*Umod*C147W^ mice Atf3 is specifically induced in the nuclei of TAL cells, likely exerting a protective role in mutant mouse kidneys. Given the primary effect of uromodulin mutation, it is likely that Atf3 is induced in TAL cells because of ER stress. Indeed, mutations in *UMOD* leading to protein misfolding and accumulation in the ER likely elicit ER and oxidative stress pathways that play a main role in the disease pathogenesis. Evidence for UPR induction in Tg^*Umod*C147W^ mice is limited in our dataset, as it was identified only in the group of male mutant mice (Reactome database, Unfolded_Protein_Response, ATF4 and PERK, data not shown). This is likely due to the fact that such cell stress pathways are expected to be increased in TAL cells only. The use of RNA from total kidneys may have masked their induction due to a dilution effect. Nevertheless, very recent data indicate UPR induction^28, 49^ and derangement of mitochondria^49^ in TAL segments of ADTKD-*UMOD* mouse models.

The induction of UPR could also be upstream of inflammation, and the coupling of these responses in specialized cells and tissues is now thought to be fundamental in the pathogenesis of inflammatory diseases^50^. In kidneys from p8 mutant mice we identified increased expression of *Ccl5* (Rantes) and *Ccl12* (MCP-5), that is a structural and functional homologue of the human *CCL2* (MCP-1)^51^. Further studies will be need to assess if these chemokines are produced by TAL epithelial cells, possibly downstream of ER stress signals, and play a role in the onset and progression of tubulointerstitial disease^52^.

In conclusion, our study identifies renal induction of inflammatory signals as an early event in the pathogenesis of ADTKD-*UMOD*. Further characterisation of these pathways is warranted to assess their relevance in the disease and as potential targets of novel therapeutic intervention.

## Materials and Methods

### Transgenic mice

A detailed description of the methods for the generation of Tg^*Umod*C147W^ and Tg^*Umod*wt^ lines was previously reported^25^. Both mutant (Cys147Trp) and wild type transgenes comprise a 2.9 Kb fragment of the mouse *Umod* gene promoter, non-coding exon 1, intron 1, the coding sequence from exon 2 to 11 and the entire 3′ UTR. An HA-tag was inserted at the uromodulin N-terminus after the leader peptide. Full constructs were injected in FVB mouse. All animal procedures were carried out at San Raffaele Scientific Institute, Milan, Italy, according to, and approved by, the San Raffaele Institutional Animal Care and Use Committee (IACUC 571). All animal studies were performed in adherence to the *Guide for the Care and Use of Laboratory Animals* as published by the US National Institutes of Health.

### Tissue collection and preparation

To collect renal tissues, mice were sacrificed by decapitation after anaesthesia with avertin. Kidneys were taken and immediately homogenized in TRIzol reagent (Invitrogen, Thermo Fisher Scientific, Waltham, MA) for RNA extraction or in appropriate lysis buffer for protein extraction. Renal tissues were fixed in 4% paraformaldehyde and paraffin-embedded for immunohistochemical and histological analysis or embedded in killik embedding medium (Bio-Optica, Milan, Italy) for immunofluorescence staining.

### Microarray Analysis

Total RNA was extracted from whole kidney with TRIzol reagent, quantified using the Nanodrop 8000 spectrophotometer (Thermo Fisher Scientific) and qualitatively analysed by agarose gel electrophoresis. Extracted RNA was treated with DNase (Qiagen, Hilden, Germany) and purified using RNeasy Mini Kit columns (Qiagen), according to the manufacture’s protocol. Four animals for each experimental group were analysed.

Transcriptional profiles were determined by using the MouseWG-6 v2.0 Expression BeadChip (Illumina, San Diego, CA). Each BeadChip can process simultaneously six samples, each one investigated for a total of 48,804 transcripts, of which 35,967 are based on the National Center for Biotechnology Information RefSeq database (Release 22) and 12,837 are based on UniGene database (Build 199). Total RNA (500 ng) was reverse transcribed into cRNA and biotin-UTP labelled using the Illumina TotalPrep RNA Amplification Kit (Applied Biosystems, Thermo Fisher Scientific) according to the manufacturer’s protocol. The cRNA was quantified with Nanodrop 8000 Spectrophotometer and its quality was evaluated with RNA 6000 NanoChip on the Bioanalyzer 2100 (Agilent Technologies, Santa Clara, CA). cRNA (1,500 ng) was then hybridized to the BeadChip Array and stained with streptavidin-Cy3. All procedures were performed following the manufacturer’s instructions. BeadChips have been imaged using the IlluminaBeadArray Reader, a two-channel 0.8-μm-resolution confocal laser scanner, and the Illumina BeadScan software. The software IlluminaGenomeStudio v.2011.1 was used to elaborate the fluorescence signal to a value whose intensity corresponds to the quantity of the respective transcript in the original sample. The same software was used to assess quality controls, including the biological specimen control, hybridization controls, signal generation controls, and negative controls. All quality controls were satisfactory. Raw data from the SampleProbeProfile txt file were exported from GenomeStudio software. Gene expression data were normalized using the cubic spline algorithm implemented in the Illumina® GenomeStudio software. An initial selection of “expressed” probes was done by filtering on the “detection *P* value” parameter. Transcripts with an intensity value significantly different from that of the background (detection *P* value < 0.01) in at least one sample of an analysed series (i.e. experimental groups taken into consideration) were considered “expressed” genes. Transcriptome data reported in this publication have been deposited in NCBI’s Gene Expression Omnibus^53^ and are accessible through GEO Series accession number GSE97093 (https://www.ncbi.nlm.nih.gov/geo/query/acc.cgi?acc=GSE97093). Subsequent multivariate analyses and differential expression were performed on the subset of expressed genes in the groups defined by the experimental design. Principal Component Analysis (PCA) was done on expressed genes using scripts in Rstudio^54^. Hierarchical clustering was carried out on the 150 genes showing the greatest inter-group variance among compared groups. Genes and samples have been clustered using Euclidean distance and average clustering algorithm (pheatmap; https://cran.r-project.org/web/packages/pheatmap/index.html). Determination of differentially expressed genes and processes was done using LIMMA Bioconductor package^55^. Pair-wise comparisons (i.e. “contrasts”) were defined among the experimental groups considered in a specific design. Benjamini Hochberg multiple comparison correction was applied to the *P* value and genes passing a cut-off adjusted *P* value < 0.05 were considered differentially expressed in the specified comparison. Gene Set Enrichment Analysis (GSEA) (http://www.broadinstitute.org/gsea/index.jsp), which identifies groups of genes enriched towards the top or bottom of a ranked list of genes based on a running sum statistics, was used to identify functionally related groups of genes whose expression pattern was correlated with the template, defined by the hallmark curated gene sets from MSigDB^56, 57^. Pathway enrichment analysis was also performed by using DAVID software (https://david.ncifcrf.gov/).

Pathways enriched during the progression of renal disease in Tg^*Umod*C147W^ mice were identified by carrying out a biological Term Enrichment Analysis using KEGG Pathways database^58^ and clusterProfiler Bioconductor package^59, 60^. We compared the prevalence of gene annotations (KEGG Pathways) among differentially expressed genes (i.e. significantly up- or down-regulated in Tg^*Umod*C147W^ female mice at 2 months of age, regardless of their fold change, and not differentially expressed at 1 month of age in sex-matched mice) to their prevalence in a background defined by all the expressed genes (as defined above). Fold enrichment was defined as the ratio gene frequency/background frequency (ratio of the number of genes annotated to a given biological term in the list of DEGs over the number of DEGs)/(ratio of the number of genes annotated to that biological term in the entire background set over the total number of genes in the background set).

### Real-time qPCR analysis

Total RNA from mouse whole kidney was extracted by homogenization in TRIzol reagent. RNA sample concentrations were determined by using Nonodrop-8000 and RNA quality was assessed on agarose gel. For each sample 1 μg of extracted RNA was reverse-transcribed using iScript kit (BioRad Laboratories, Hercules, CA) according to the manufacturer’s protocol. Expression of target genes was analysed by RT-qPCR on LightCycler 480 (Roche, Basel, Switzerland) using qPCR Core kit for SYBR Assay (Eurogentec, Liège, Belgium). Specific primers were designed by using Primer 3 (a list of all primers used in this study is reported in Supplementary Table 1) and the amplification efficiency for each couple of primers was determined by dilution curves. Expression of genes of interest was normalized to *Hprt1*. The relative mRNA expression was calculated following the ΔΔCT method^61^.

### Immunohistochemistry and histology

Immunohistochemistry was performed on 5 µm-thick kidney sections using standard procedures (primary antibodies are listed below) followed by a counterstaining with hematoxylin. Routine staining (Periodic acid–Schiff, PAS; Acid Fuchsin Orange G, AFOG), were performed on kidney slices (5 µm-thick) according to standard protocols. For both immunohistochemistry and histological analysis, sections were viewed under a Zeiss Axioscope 40FL microscope (Carl Zeiss, Oberkochen, Germany), equipped with AxioCam MRc5 digital video camera. Images of kidney slices from Tg^*Umod*C147W^ and Tg^*Umod*wt^ mice were recorded using identical parameters with AxioVision software 4.3 (Carl Zeiss). Quantification of histological features was performed on stained renal sections by an observer unaware of the mouse genotype. Mesangial expansion and mesangial hypercellularity were assessed semiquantitatively (0 = absent; 1 = 1–50%; 2 = 51–100%). Interstitial inflammation and fibrosis, presence of tubular casts and tubular dilation were assessed semiquantitatively (0 = absent; 1 = 1–30%; 2 = 31–60%; 3 = 61–100%). Presence of focal/segmental glomerulosclerosis was quantitatively expressed as % of the total number of glomeruli (30 glomeruli for each sample).

### Immunofluorescence staining

Immunofluorescence was carried out using standard protocol on 7 µm-thick kidney sections. Briefly, after 1 h in blocking solution, slides were incubated over-night with specific primary antibody (see below) and then with appropriate AlexaFluor-labeled secondary antibody (Life Technologies, Thermo Fisher Scientific). All slides were viewed under a DM 5000B fluorescence upright microscope (Leica DFC480 camera, Leica DFC Twain Software, 40X/0.75 lens) (Leica Microsystems, Wetzlar, Germany) or under an UltraVIEW ERS spinning disk confocal microscope (UltraVIEW ERS-Imaging Suite Software, Zeiss 63X/1.4; PerkinElmer Life and Analytical Sciences Boston, MA). Identical acquisition parameters were ensured for the visualization of the same antibody in different kidney sections. All images were imported in Photoshop CS (Adobe Systems, Mountain View, CA) and adjusted for brightness and contrast.

### Western blot analysis

Mouse renal tissues were homogenized on ice immediately after explants in the following buffer (to detect transgenic uromodulin and Kim1): NaCl 150 mM, N-octylglucoside 60 mM, Protease-Inhibitor Cocktail 1:1,000 (Sigma-Aldrich, St. Louis, MO) and Tris-HCl 20 mM, pH 7.4 or in the following buffer (to detect Atf3 and Lcn2): Urea 8 M, NaCl 500 mM, EDTA 0.1 mM, EGTA 0.1 mM, Nonidet P40 0.1% (v/v), DTT 1 mM, Protease-Inhibitor Cocktail 1:1,000 (Sigma-Aldrich) and HEPES 10 mM, pH 7.9. Protein lysates were separated on an 8% (transgenic uromodulin and Kim1) or 12% (Atf3 and Lcn2) SDS-PAGE gel in reducing condition and transferred onto nitrocellulose membrane (GE Healthcare, Chicago, IL). Immunoblot was performed following standard protocols. Quantification of the optical band densities was performed using the gel analysis option of ImageJ software^62^. The optical density of proteins of interest was normalized to the one of β-Actin or Gapdh (loading controls) run on the same gel.

### Antibodies

The following antibodies were used for immunohistochemical, immunofluorescence and Western blot analyses: sheep polyclonal antibody against uromodulin (ab9029, Abcam, Cambridge, UK) (1:200 for IF and for IHC); rat monoclonal antibody against HA (#11 867 423 001, Roche) (1:500 for IF and 1:1,000 for WB); rabbit polyclonal antibody against calreticulin (C4606, Sigma-Aldrich) (1:500 for IF); rat antibody against mouse F4/80 (MCA497GA, Serotec, BioRad)(1:50 for IF and IHC); rabbit polyclonal antibody against Atf3 (sc-188, Santa Cruz Biotechnology, Dallas, TX) (1:50 for IF and 1:500 for WB); goat polyclonal against Lipocalin 2/NGAL (AF1857, R & D Systems, Bio-Techne, Minneapolis, MN) (1:500 for WB); rabbit polyclonal against Kim1 (TIM-1) (NBP1-76701, Novus Biologicals, Bio-Techne) (1:500 for WB); mouse monoclonal antibody against β-Actin (A2228, Sigma-Aldrich) (1:20,000 for WB); mouse monoclonal antibody against Gapdh (sc-32233, Santa Cruz Biotechnology) (1:20,000 for WB).

### Statistical analysis

Data are means ± standard deviation (s.d.) or standard error of mean (s.e.m.). Comparisons between groups were performed using two-tailed unpaired Student’s *t*-test, or two-tailed non-parametric Mann-Whitney test. Significance level was set to *P* < 0.05.

## Electronic supplementary material

Supplementary Information
