# Supplementary material for: Early involvement of cellular stress and inflammatory signals in the pathogenesis of tubulointerstitial kidney disease due to UMOD mutations
Source: Sci Rep. 2017 Aug 7;7:7383. doi: 10.1038/s41598-017-07804-6 (PMC5547146; doi:10.1038/s41598-017-07804-6)
Supplement: Supplementary file 1 — Supplementary Information [file 41598_2017_7804_MOESM1_ESM.pdf]

# Early involvement of cellular stress and inflammatory signals in the pathogenesis of tubulointerstitial kidney disease due to *UMOD* mutations

Matteo Trudu[1], Celine Schaeffer[1], Michela Riba[2], Masami Ikehata[3,4], Paola Brambilla[5], Piergiorgio Messa[3,4], Filippo Martinelli-Boneschi[5], Maria Pia Rastaldi[3], Luca Rampoldi[1*]

## *SUPPLEMENTARY INFORMATION*

***Supplementary Figures 1-6, Supplementary Table 1***

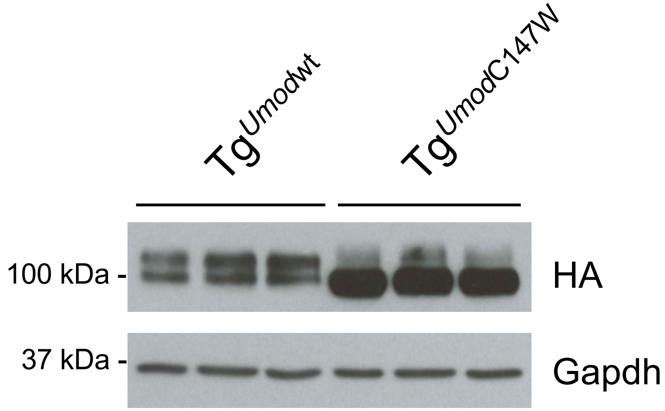


**Supplementary Figure 1**. Western blot analysis showing expression of HA-tagged transgenic uromodulin in kidneys of 1 month-old $Tg^{\mathit{Umod}\mathrm{wt}}$ and $Tg^{\mathit{Umod}\mathrm{C147W}}$ mice. The figure shows cropped blot images (full blots are reported in **Supplementary Figure 6**). Mutant uromodulin is strongly retained in the ER, as demonstrated by the accumulation of the lower molecular weight isoform, corresponding to uromodulin ER precursor. Gapdh was used as loading control.

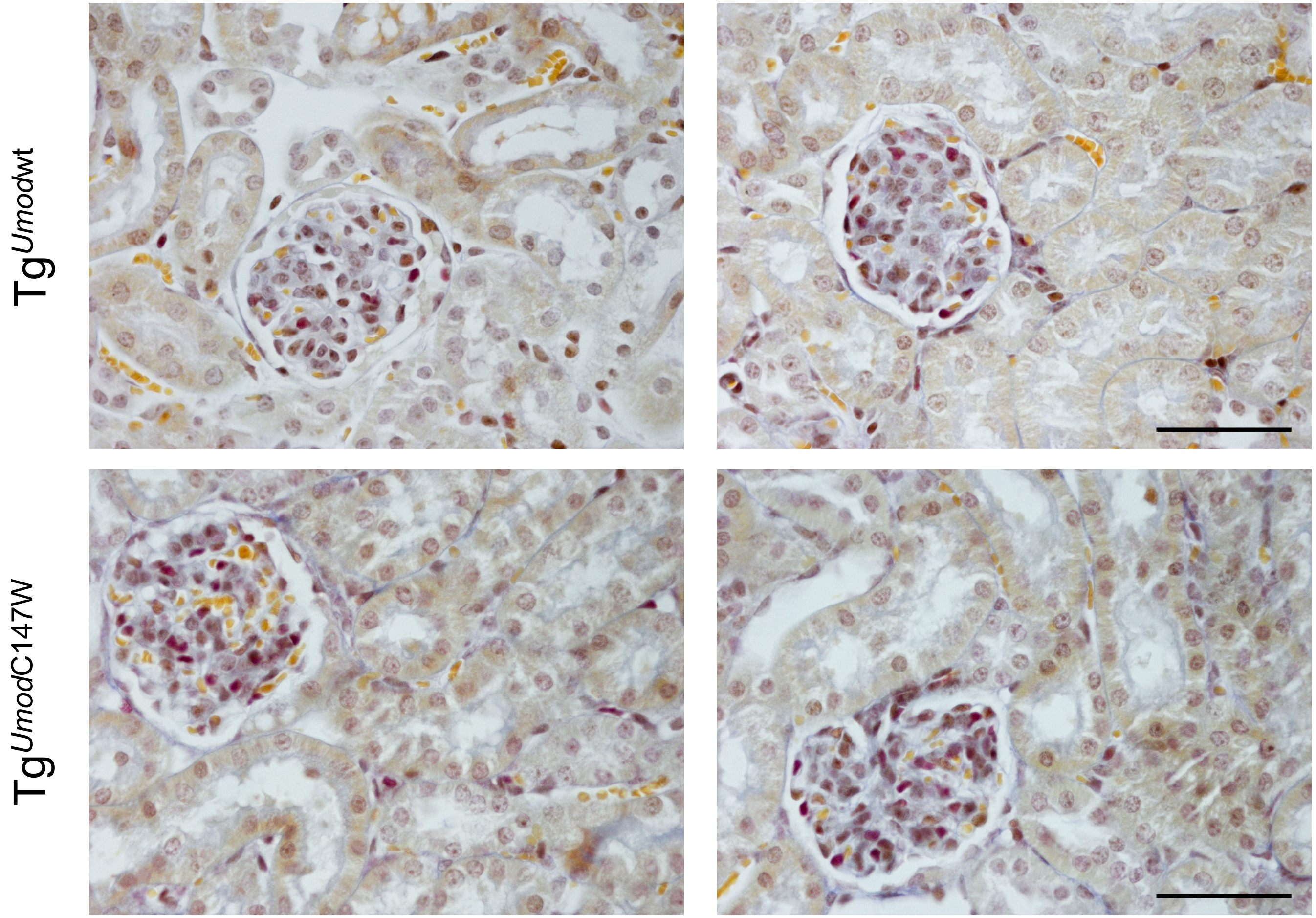


**Supplementary Figure 2.** Representative histological images of glomeruli in kidneys of 1 month-old $Tg^{UmodC147W}$ and $Tg^{Umodwt}$ (AFOG; scale bar 50 μm). Quantification of mesangial hypercellularity, mesangial matrix expansion and focal/segmental glomerulosclerosis showed no difference between $Tg^{UmodC147W}$ and $Tg^{Umodwt}$ mice (data not shown, n = 9 $Tg^{Umodwt}$ and 6 $Tg^{UmodC147W}$).

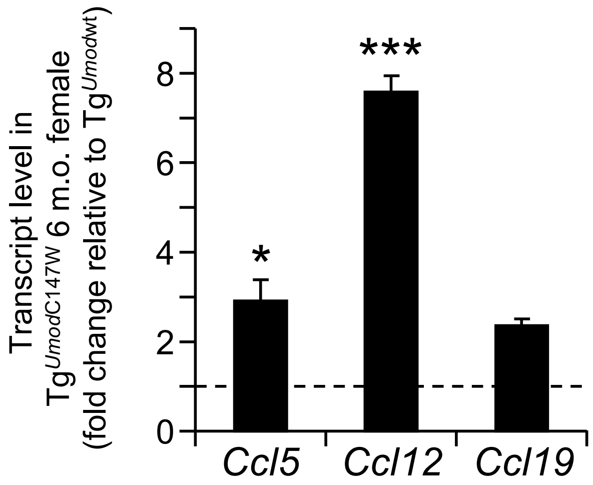


**Supplementary Figure 3.** Analysis by real-time RT-qPCR of the expression of selected chemokines in 6 month-old $Tg^{\mathit{Umod}\mathrm{C147W}}$ mice relative to age- and sex-matched $Tg^{\mathit{Umod}\mathrm{wt}}$ (n = 5/group). Data are expressed as mean ± s.e.m. *$P$ < 0.05; ***$P$ < 0.001 (unpaired *t*-test).

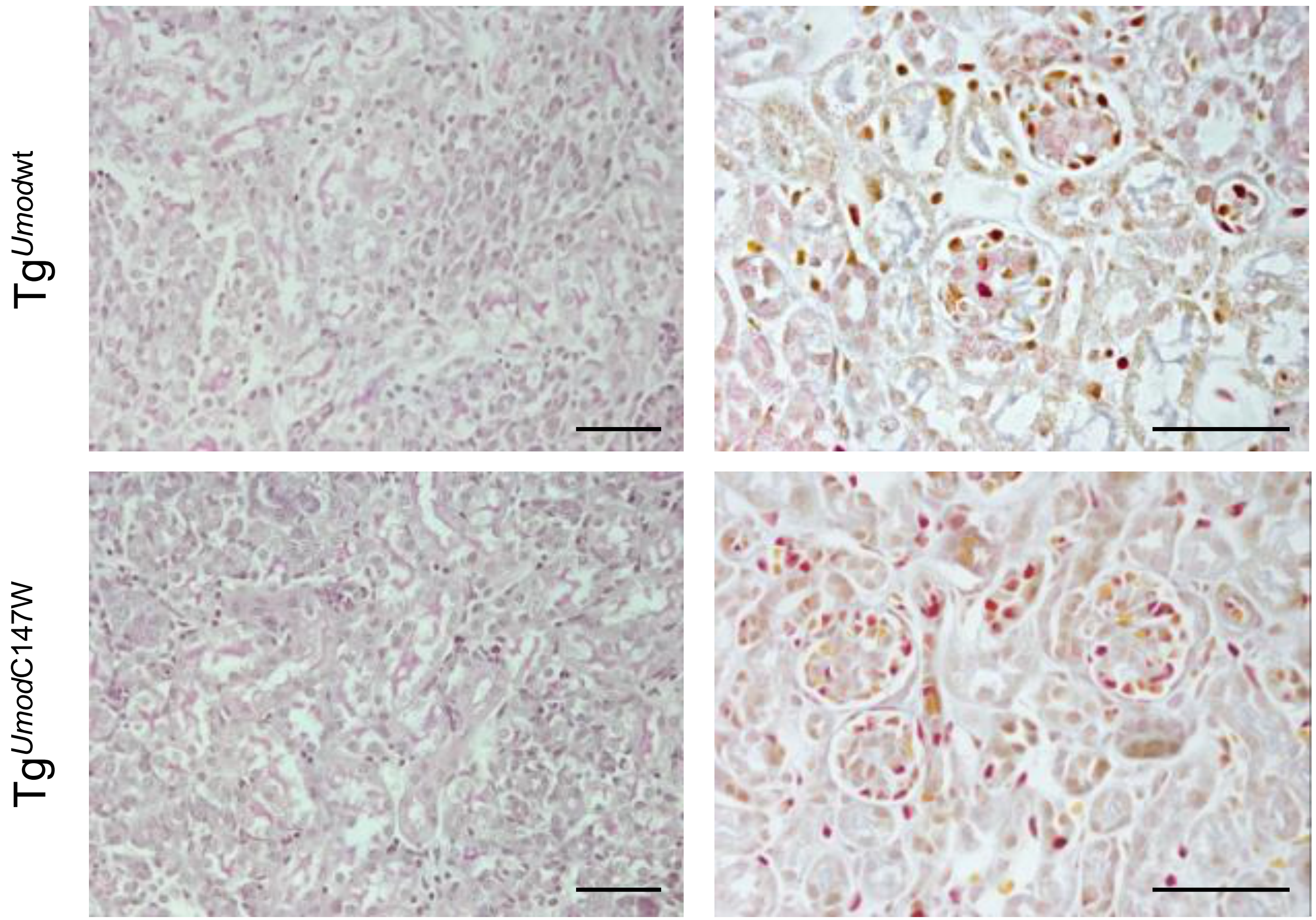


**Supplementary Figure 4.** Representative histological images of tubular architecture and glomeruli in kidneys of $Tg^{\mathit{Umod}\mathrm{C147W}}$ and $Tg^{\mathit{Umod}\mathrm{wt}}$ mice at p8 (PAS, left panels, and AFOG, right panels; scale bar 50 µm). Quantification of tubular casts, tubular damage, interstitial inflammation, interstitial fibrosis, mesangial hypercellularity, mesangial matrix expansion and focal/segmental glomerulosclerosis showed no difference between $Tg^{\mathit{Umod}\mathrm{C147W}}$ and $Tg^{\mathit{Umod}\mathrm{wt}}$ mice (data not shown, n = 4/group).

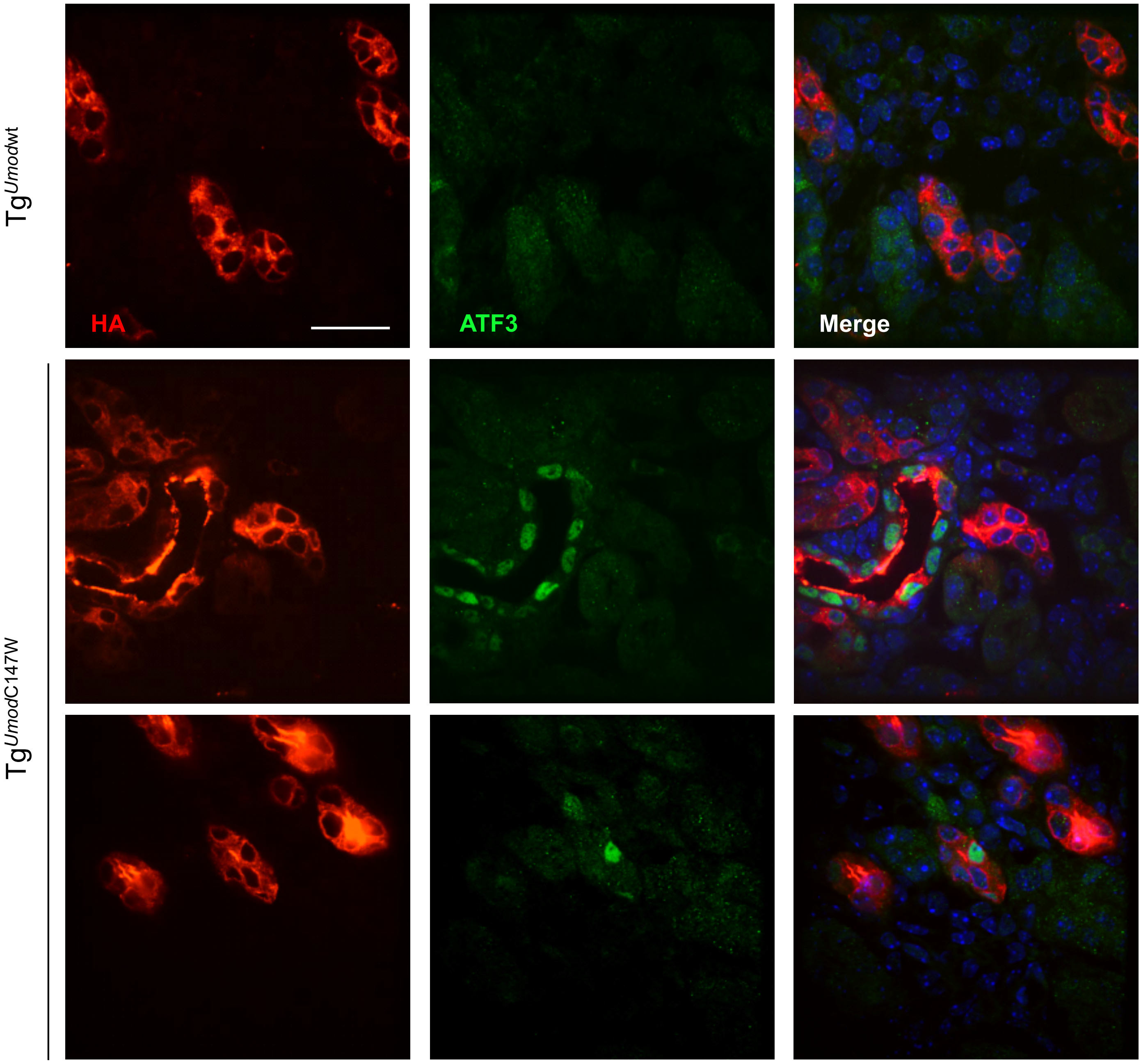


**Supplementary Figure 5.** Expression of Atf3 in the kidneys of p8 transgenic mice. Immunofluorescence analysis reveals that Atf3 is specifically detected in nuclei of TAL segments in kidneys of Tg$^{Umod C147W}$ mice (scale bar 15 µm).

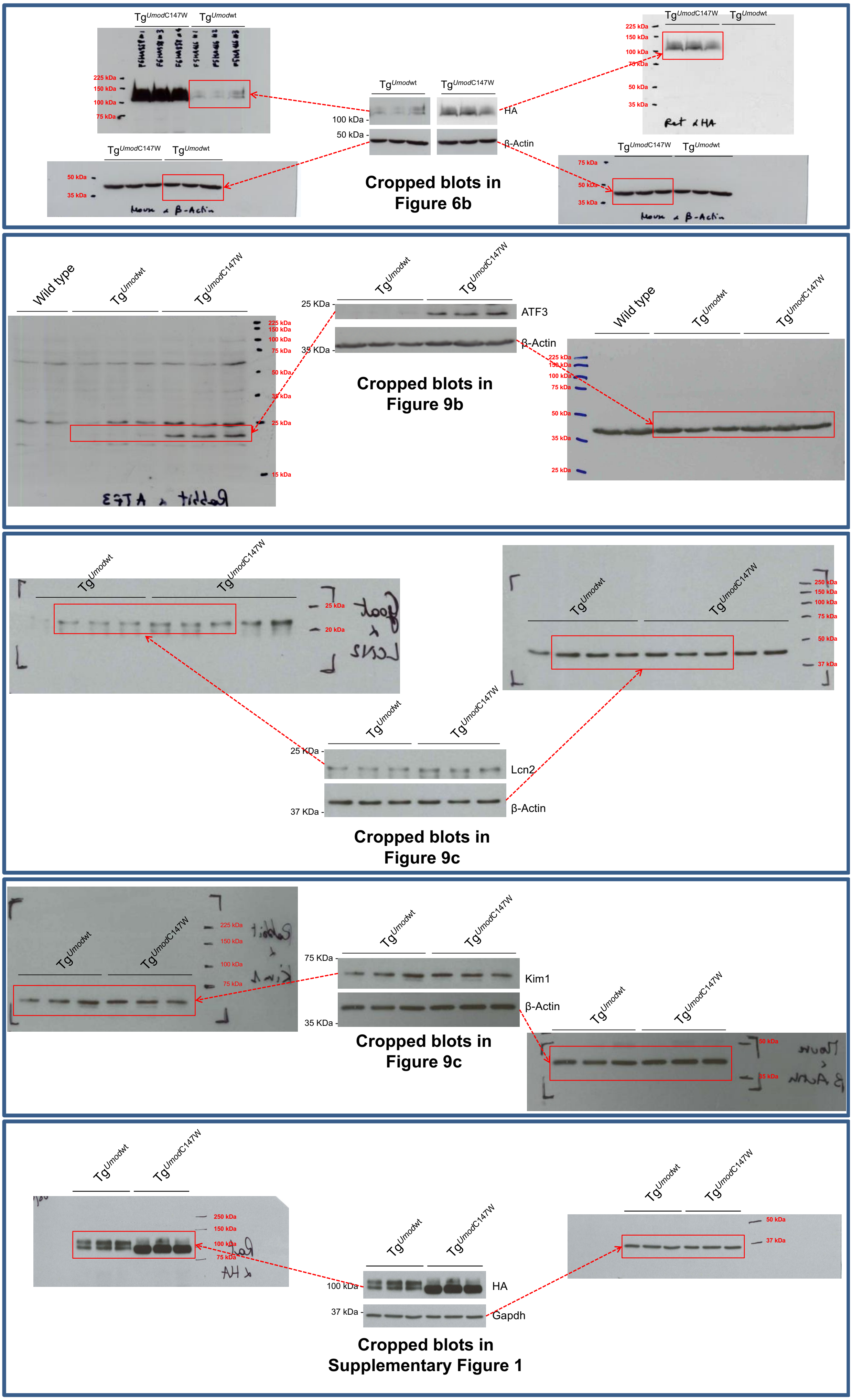


**Supplementary Figure 6.** Full image of blots shown as cropped images in **Figure 6b**, **Figure 9b**, **Figure 9c**, **Supplementary Figure 1**. The red boxes indicate the cropped images.

**Supplementary Table 1.** List of primers used for SYBR Green real-time RT-qPCR.

| **Target gene** | **Forward primer (5'-3')** | **Reverse primer (5'-3')** |
|---|---|---|
| ***Ccl5*** | GTGCCCACGTCAAGGAGTATT | CCCACTTCTTCTCTGGGTTGG |
| ***Ccl12*** | CATCAGTCCTCAGGTATTGGCTGGA | CTTGGGGTCAGCACAGATCTCCTT |
| ***Ccl19*** | CTGGCCTTCAGCCTGCTGGT | TGCGATCCACCCAGGGCTGG |
| ***Tgfb1*** | CCCGCGTGCTAATGGTGGACC | TGCACGGGACAGCAATGGGG |
| ***Vim*** | GGATCAGCTCACCAACGACA | GGTCAAGACGTGCCAGAGAA |
| ***Col6a1*** | ACCGACTGCGCCATTAAGAA | GTCGGTCACCACGATCAAGT |
| ***Acta2*** | GCTACGAACTGCCTGACGG | GCTGTTATAGGTGGTTTCGTGGA |
| ***Acox3*** | GCTCACTTCACAAGCCCTCT | TAGCAAACAAGCCAGCGGTA |
| ***Ehhadh*** | GCCATAGTGATCTGTGGAGCA | ACCACTGGCTTCTGGTATCG |
| ***Cyp4b1*** | CCAGCTCAGCAAGCCAGTAA | GTGGGTCAAAGACCTCTGGG |
| ***Col1a1*** | CTGACGCATGGCCAAGAAGA | ATACCTCGGGTTTCCACGTC |
| ***Ntn4*** | AGGATTTTCTGCCCTCCGAC | ATGGGAGCCTTTGTCGTGG |
| ***Panx1*** | GCTCATCTCGCTGGCCTTCGC | AATCCACAAAGGCAGCCTGTCGC |
| ***Slc13a3*** | TATGGTGGAATGAGCTGGAGA | GGCAAACGTGCAGAACAAGAT |
| ***Slc25a17*** | GCCTCTGTGCTGTCCTACG | ACCTGAAGCCGAAGTCTAGC |
| ***Cd5*** | CCCTTGCCAATTCGATGGGA | GGGCTGGAAATCAGAGCAGA |
| ***Cd19*** | ACCAGTTGGCAGGATGATGG | GCTGAGGAGCTGCATAGAGG |
| ***Ptprc*** | GGAGACCAGGAAGTCTGTGC | GTTCTGGGCTCCTTCCTCTT |
| ***Cd68*** | CTGACAAGGGACACTTCGGG | AGGCCAATGATGAGAGGCAG |
| ***Fut4*** | GAGGTGGGTGTGGATGAACT | GTTGGATCGCTCCTGGAATA |
| ***Lcn2*** | TCCCCCTGCAGCCAGACTTCC | AGTAGCGACAGCCCTGGTCCTG |
| ***Havcr1*** | TTGGCATCTGCATCGCAGCCC | GGGAATGCACAACCGCTGCGT |
| ***Atf3*** | CAGAAGTCAGTGCGACCGCC | TCGCCGCCTCCTTTTCCTCTCA |
| ***Hprt1*** | ACATTGTGGCCCTCTGTGTG | TTATGTCCCCCGTTGACTGA |
